# MYC: a multipurpose oncogene with prognostic and therapeutic implications in blood malignancies

**DOI:** 10.1186/s13045-021-01111-4

**Published:** 2021-08-09

**Authors:** Seyed Esmaeil Ahmadi, Samira Rahimi, Bahman Zarandi, Rouzbeh Chegeni, Majid Safa

**Affiliations:** 1 Department of Hematology and Blood Banking, Faculty of Allied Medicine, Iran University of Medical Sciences, Tehran, Iran; 2 Medical Laboratory Sciences Program, College of Health and Human Sciences, Northern Illinois University, DeKalb, IL USA; 3 Cellular and Molecular Research Center, Iran University of Medical Sciences, Tehran, Iran

**Keywords:** MYC, Oncogene, Regulation, Cell cycle, Apoptosis, DNA damage response, Prognostic importance, Therapeutic implications, Hematological malignancies

## Abstract

MYC oncogene is a transcription factor with a wide array of functions affecting cellular activities such as cell cycle, apoptosis, DNA damage response, and hematopoiesis. Due to the multi-functionality of MYC, its expression is regulated at multiple levels. Deregulation of this oncogene can give rise to a variety of cancers. In this review, MYC regulation and the mechanisms by which MYC adjusts cellular functions and its implication in hematologic malignancies are summarized. Further, we also discuss potential inhibitors of MYC that could be beneficial for treating hematologic malignancies.

## Introduction

MYC (mostly referred to as c-Myc) is a super-transcription factor encoded by the MYC gene located at chromosome 8 q24.21 [1]. The MYC oncoproteins (C-myc, N-myc, and L-myc) controls the transcription of nearly 15% of expressed genes [2]. MYC's main downstream mediators, including those participating in ribosome biogenesis, mRNA translation, cell-cycle regulation, and stress responses, impact a vast range of biological events, such as proliferation, differentiation, survival, programmed cell death, and immune regulation [2, 3].

**Fig. 1:**
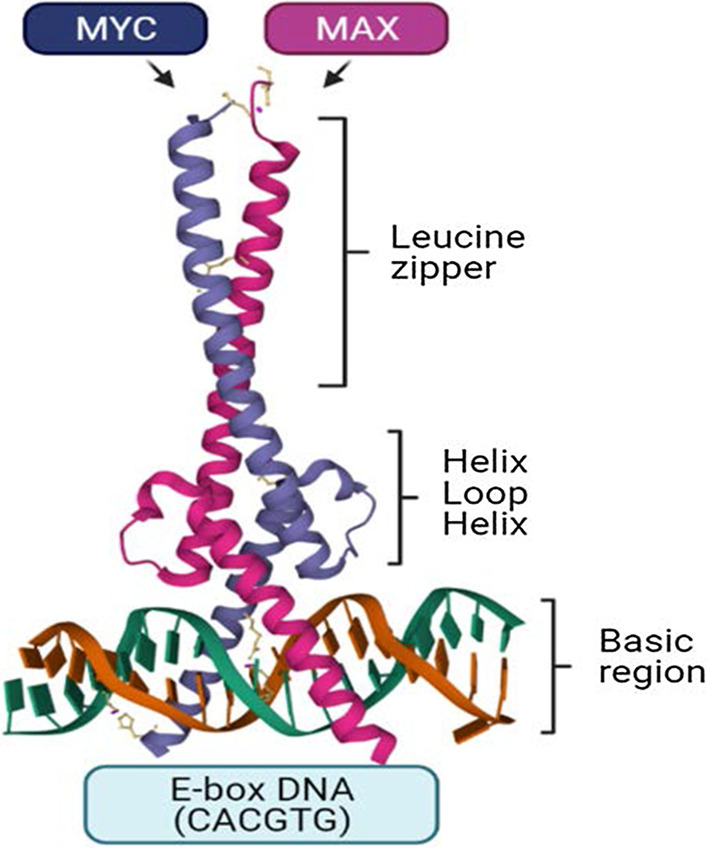
Crystal structure of MYC/MAX heterodimer. MYC usually forms as a heterodimer with MAX (MYC/MAX) to bind to DNA in E-box region (CACGTG). This structure mainly contains the basic-region (BR), helix-loop-helix (HLH), and leucine-zipper (LZ), which are required for DNA binding

**Fig. 2:**
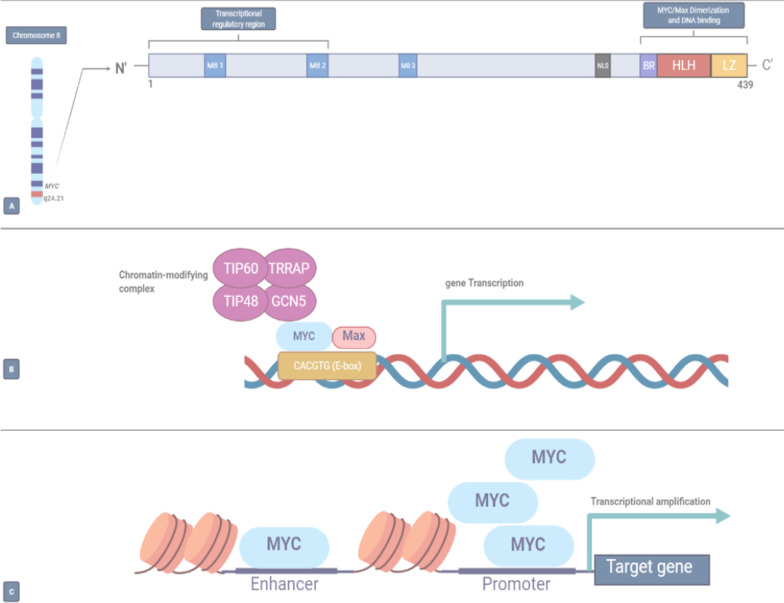
Schematics of MYC protein and its transcriptional activity. **A**: MYC gene on chromosome 8 alongside MYC protein (439 aa) that mainly contains the basic-region (BR), helix-loop-helix (HLH), and leucine-zipper (LZ) at C-terminal, and three extremely conserved regions called MYC boxes 1–3 (MB 1–3) at the N-terminal. **B**: The chromatin-modifying complex consisting of TIP60, TRRAP, TIP48, and GCN5 recruited by MYC/Max heterodimer propels transcription through binding to the E-box DNA region (CACGTG) within the regulatory domain of target genes. **C**: the accumulation of MYC at the promoter sequences of target genes increases the transcriptional activity. *NLS* nuclear localization sequence

There is a high level of architectural homology in the motifs at the flanked domains of the MYC family members, including the basic-region (BR), helix-loop-helix (HLH), and leucine-zipper (LZ) in C-terminal, and three extremely conserved regions called MYC boxes 1–3 (MB 1–3) at the N-terminal [3–5]. MYC creates a heterodimer with its co-factor, Max (MYC/Max), via BR, HLH, and LZ motifs requisite for DNA–protein interactions (Fig. 1) [3–5]. The chromatin-modifying complex consisting of TIP60, TRRAP, TIP48, and GCN5 recruited by MYC/Max heterodimer propels transcription through binding to the E-box DNA region (CACGTG) within the regulatory domain of target genes [3–5]. Accumulation of MYC at the promoter sequences of target genes can also augment the transcriptional activity of genes (Fig. 2) [6].

The MYC expression pattern is tightly regulated in normal conditions, though MYC is often dysregulated in cancers. Retroviral integration, chromosomal rearrangements, activation of super-enhancers of its gene, and mutations in signaling pathways related to MYC can promote MYC's instability and overexpression [3]. The MYC expression is highly controlled at several levels, including transcription (initiation and elongation), mRNA stability, translation, and post-translation (protein stability). MYC is a very short-lived protein with a half-life of about 20–30 min because of quick turnover through the ubiquitin–proteasome system [7]. Consequently, the MYC protein level is strongly controlled by ubiquitin–proteasome degradation.

The pivotal role of MYC in the cell cycle regulation and the proliferation rate has been deeply investigated in several studies. Reduced need for growth factors, increased cell division, and size can be seen in response to transfection or transduction with MYC [8–10]. Entering and exiting cell-cycle is achievable by decreasing or increasing MYC expression [11, 12]. After mitogenic stimulation of MYC expression, which is undetectable in quiescent cells, MYC increases rapidly and mediates cell entry to the G1 phase. This is followed by a decrease in MYC mRNA and protein levels [13]. A better understanding of cell-cycle regulation by MYC helps find novel therapeutic approaches to target the MYC.

The role of MYC in cell damage has been investigated in numerous studies. In DNA damage caused by UV irradiation or other agents, MYC levels are decreased through different mechanisms, including alternation in MYC transcription and protein turnover [14–16]. The results of several studies exhibit that decreased levels of MYC are seen as a DNA damage response (DDR) [15, 17, 18]. A decreased MYC levels and accumulation of p53 in DDR is a normal response to regulating cell damage [14]. MYC promotes apoptosis via increasing the p53 levels indirectly, in turn, p53 suppresses MYC expression. DNA repair inhibition, ROS generation, and increased replication stress are among the MYC-induced DDR mechanisms [19]. In cancer however, this fine-tuned interplay between p53 and MYC is mostly deregulated.

The first oncogene reported to induce apoptosis was MYC [20]. A well-known fundamental function of MYC is induction of apoptosis. MYC transcription factor has a dual role in tumor cells. It can activate and repress various downstream pathways that can induce proliferation or apoptosis [6]. Apoptosis has a role in physiological processes, such as embryonic development, tissue morphogenesis cellular hemostasis life. Hence, MYC-induced apoptosis indicates this transcription factor's normal function in controlling cell death [21]. Indeed, MYC exerts a safeguard mechanism by induction of apoptosis. It should be noted that a higher level of MYC is required for apoptosis compared to the concentrations needed to trigger cell proliferation, indicating that under normal conditions, cells are able to proliferate [22].

The MYC is a “global” transcription factor contributing to various cellular processes, one of which is hematopoiesis. In the bone marrow (BM) of adults, 300 million cells are produced every minute [23]. Regulation of hematopoiesis requires cell–cell interactions, cytokines, and coordinated activity of transcription factors. Studies have revealed that MYC has a significant role in nearly every step of the way [23, 24].

Uncontrolled MYC expression is observed in human leukemias and lymphomas. Generally, MYC overexpression does not stem from point mutations in the gene [25–27]. Rather in hematological malignancies such as acute lymphoblastic leukemia (ALL), chronic lymphocytic leukemia (CLL), and myeloid neoplasms, overexpression is mainly due to the gene amplification, chromosomal translocations, and dysregulation at the transcriptional level [28]. Overall, given MYC’s functions, it is not surprising that deregulation and deletion of MYC can contribute to tumorigenesis, particularly in hematological cells.

Aberrant MYC expression usually confers a poor prognosis. Targeting the MYC family, especially MYC, is of utmost significance in identifying treatment options for hematological malignancies [29]. Here, we explain the role of MYC in various cellular functions, including cell cycle, MYC-mediated DDR, and apoptosis, as well as MYC regulatory processes. In particular, different types of hematological malignancies and their association with MYC deregulation have been thoroughly discussed in this review along with the effects of various MYC inhibitors.

## MYC regulation

MYC regulation and transcriptional activity are critical to maintaining normal cellular processes such as cell growth, differentiation, and programmed cell death. Deregulation of MYC oncogene has been shown to contribute to more than half of human cancers [4, 30].

The mechanisms that control MYC transcription are complex. Several promoters of MYC such as P0, P1, P2, P3, and initiation regions are involved. Multiple signals, transcription factors, and chromatin components have a role in the regulation of MYC mRNA levels [31, 32]. The nuclear factor of activated T cells (NFAT) family of transcription factors includes four Ca^2+^-regulated members (NFAT1-NFAT4) initially discovered in T lymphocyte as transcriptional activators of interleukin 2 [33]. Previous studies indicate that NFAT1/2 can regulate MYC gene expression by binding to specific sequence elements within the proximal MYC promoter [34]. Mognol and et al. demonstrated that the Ca^2+^\calcineurin\NFAT1 signaling pathway in mouse T lymphocyte regulates MYC expression, the difference is that NFAT1 binds to the distal site of the MYC promoter. Since the lack of NFAT1 in the studied cells shows decreased levels of MYC, NFAT1 is known as a positive regulator of MYC expression [35].

In addition to transcriptional regulation, MYC stability and activity are regulated by several post-translational modifications (PTM), such as phosphorylation, acetylation, methylation, ubiquitination, sumoylation, and glycosylation. There are multiple domains in MYC that different proteins interact with. The transactivation domain (TAD), is a 143 amino acid acidic domain localized at the N-terminus. It contains two conserved regions, Myc box (MB) I and II, mainly required for MYC regulation and cofactor recruitment, respectively [36, 37]. MYC contains two phosphorylation sites near its within MB I, Threonine 58 (Thr-58), and Serine 62 (Ser-62), which are highly conserved across all mammalian MYC isoforms [38, 39].

Phosphorylation of Ser-62 MYC by extracellular receptor kinase (ERK) and cyclin-dependent protein kinase 2 (CDK2) lead to stabilizing MYC whereas Thr-58 phosphorylation by glycogen synthase kinase (GSK-3β) results in degradation of MYC through the ubiquitin–proteasome pathway [40]. It has been shown that both Raf\MEK\ERK kinase cascade and the phosphoinositide 3-kinases (PI3K) \Akt signaling pathway significantly elevate the half-life of MYC through negative feedback. Mitogenic stimulation can promote production and stability of Myc and activation of Ras. Ras increases MYC protein stability by ERK-mediated phosphorylation of Ser-62 [40, 41]. Ras induces activation of PI3K\Akt cascade that leads to preventing phosphorylation of Thr-58 by suppressing GSK-3β and stabilizing and elevating MYC protein levels. During the late G1 phase of the cell cycle, reduced Ras activity, leads to Akt signaling downregulation, which results in destabilization and degradation of MYC [42]. Studies show that interaction between Ser-62 and Thr-58 play a vital role in regulating MYC expression during induced cell proliferation.

Bromodomain protein 4 (BRD4) is an epigenetic and transcriptional regulator with intrinsic histone acetyltransferase (HAT) and\or kinase activities localized at its carboxy-terminal and amino-terminal domains, respectively [43]. Similar to GSK-3β, BRD4 directly interacts with Myc and phosphorylates it at Thr-58, resulting in Myc destabilization. GSK-3β is mostly cytoplasmic and translocates to the nucleus in response to inducing extrinsic signaling, but BRD4 is predominantly in nucleus thus, it is more likely that BRD4 plays a more critical role in maintaining hemostatic levels of Myc. Moreover, BRD4, ERK1, and Myc form a trimeric complex and regulator network to sustain hemostatic levels of Myc. On the contrary, Myc can suppress the HAT activity of BRD4 and thereby regulate BRD4 function while ERK1 inhibits the BRD4 kinase activity [44].

The Ras\Raf signaling cascade has an important role in the regulation of the MYC promoter. Small GTP-binding protein Ras promotes MYC expression by inducing the Raf\MAPK\MEK pathway. Platelet-derived growth factor (PDGF) receptors and Src kinase also can augment the activity of Ras proto-oncogene, which results in activating the mitogen-activated protein kinase (MAPK) pathway [45]. However, both PDGF receptors and Src mediate the induction of MYC expression independently of Ras. Indeed, in response to PDGF, Src activates a signaling pathway known as the Src pathway that culminates in the transcription of MYC. Src phosphorylates Vav2 mediator, resulting in the activation of Rho proteins such as Rho, Rac, and cell division complex 42 (cdc42). Evidence shows that activated Rac highly stimulates MYC promoter and increases MYC mRNA levels in NIH3T3 cells. Rho and cdc42 also induce MYC promoter and MYC expression [46].

Protein phosphatase 2A (PP2A) is a major substrate-specific Serine/Threonine phosphatase that regulates MYC protein levels. PP2A is a heterotrimeric protein composed of a scaffold A subunit, catalytic C subunit, and a third highly variable regulatory B subunit [47, 48]. Structural A and catalytic C subunits exist in two isoforms, α or β. Regulatory B subunits fall into more than 23 isoforms belonging to four unrelated families named B\B55, B′/B56, B″, and B‴. B56 subunits include α, β, γ, δ, and ε isoforms. B56α is the only B subunit able to negatively regulates MYC protein stability and function [49, 50]. PP2A complex targeting the MYC protein phosphorylated at Ser-62 and Thr-58, dephosphorylates Ser-62 residue and regulate MYC turnover through ubiquitin-mediated proteasomal degradation [50]. Moreover, PP2A, which contains the B56α subunit, also can activate GSK-3β by dephosphorylating it [51].

The Pin1 prolyl isomerase is an essential controller of the phosphorylation signaling pathway that explicitly recognizes and isomerize the phosphorylated Serine\Threonine-Proline (phospho(p)Ser\Thr-Pro) motifs [52]. Pin1 can also convert the cis conformation to trans. The double-phosphorylated MYC (pThr-58 and pSer-62) is recognized and undergoes isomerization by Pin1, which catalyzes conversion of Pro-63 Myc to the trans conformation. This isomerization at Pro-63 Myc makes it an ideal substrate for PP2A-B56α to remove stabilizing Ser-62 residue and targets pThr-58 Myc for ubiquitin-mediated proteasomal degradation by the E3 ubiquitin ligases [53–55]. Furthermore, the MYC can be a substrate for Pin1 directly. WW phospho-binding domain of Pin1 is required for interaction with MYC, which recognizes phosphorylated sites. Phosphorylation at Thr-58 and Ser-62 residues can affect Pin1 interaction with the MBI site of MYC. The evidence indicates that the role of Thr-58 compared with Ser-62 is more critical for Pin1 binding to MYC [56]. Pin1 also affects pSer-62 MYC through stabilizing Pro-63 in the cis conformation. This results in protecting Ser-62 phosphate from PP2A-B56α-mediated dephosphorylation. This function of Pin1increases Myc stability, prolongs its interaction with DNA, and alters its transcription activity [57]. Thus, Pin1 could have a dual function by catalyzing the conformational change between cis and trans.

The main mechanism for controlling Myc family protein turnover is ubiquitin-mediated degradation by different E3 ubiquitin ligases. MYC is poly-ubiquitinylated by several E3 ubiquitin ligases, including Fbw7, Skp2, TRUSS, HectH9, TRIM32, and CHIP [58–63]. Fbw7 (also named hCdc4 or hSel 10) is a well-known E3 ubiquitin ligase. It is a member of the F-box proteins family that are components of SCF-type (Skp-Cullin-F box) ubiquitin ligase [64]. The Fbw7 human gene encodes three isoforms (Fbw7α, Fbw7β, and Fbw7γ), by alternative splicing. These isoforms are distinct in their subcellular localization (Fbw7α: nucleoplasmic, Fbw7β: cytoplasmic, Fbw7γ: nucleolar). Among them, both the Fbw7α and Fbw7γ isoforms are involved in regulating Myc protein turnover [65, 66]. MYC phosphorylation at Thr-58 and Ser-62 is required for Fbw7 to regulate MYC stability. Fbw7 recognizes the phospho-degron sequence that includes Thr-58 and Ser-62 within MBI. They control the Fbw7-mediated turnover of MYC. When Pin1 and PP2A-B56α dephosphorylate Ser-62, Fbw7 E3 ligase recognizes pThr-58 and mediates degradation of MYC by 26S proteasome [54, 59].

The multi-domain scaffold protein Axin1 stimulates formation of a complex between GSK-3β, PP2A-B56α, Pin1 and MYC. This complex can undergo ubiquitin-mediated degradation to suppress MYC transcriptional activity. Chromatin immunoprecipitation detects Axin1 on Myc promoter along with Fbw7, GSK-3β, PP2A-B56α, Pin1 complex and parts of 26S proteasome [55, 57].

F-box protein Skp2 is another E3 ubiquitin ligase and belongs to the Cullin-RING ligase that is identified for MYC ubiquitination and degradation. Skp2 interacts with two conserved and functionally vital regions of the MYC, basic-helix-loop-helix-leucine zipper (bHLHZ) motif (amino acids 379–418) and MBII (amino acids 129–147). During G1 to S phase transition this stimulates MYC degradation [60, 67]. Unlike Fbw7, which is associated with the MBI domain of MYC, Skp2-mediated ubiquitination is phosphorylation independent [67]. Although Skp2 reduces MYC protein stability and induce its degradation, this complex has the opposite effect on MYC transcriptional activity, which means that Skp2 as a cofactor of MYC promotes its transcriptional activity [60].

In addition to Fwb7 and Skp2 as the two main pathways of ubiquitin-mediated degradation of MYC, the other pathways exist for Myc degradation. Chung and colleagues first reported Romo1 (Reactive oxygen species modulator 1) in 2006 as a protein that enhances cellular ROS levels [68]. Romo1 is located in the mitochondrial membrane and induces ROS release produced by complex III of the mitochondrial electron transport chain into the cytosol [69]. Indeed, Romo1 can cause cytoplasmic translocation of Skp2 and Myc promoting its ubiquitination and degradation. Romo1\ROS\Skp2 is another pathway involved in Myc turnover. Romo1 also can promote Skp2\Myc interaction and Myc ubiquitination. Lee et al. demonstrated in a negative feedback loop, Myc stimulates Romo1 expression to increase cellular ROS levels. ROS in turn enhances cytoplasmic translocation of Skp2, which results in Myc ubiquitination and degradation [70].

Li et al. studies indicate the 11S proteasome activator REGγ as a novel ubiquitination-independent pathway to promote MYC turnover [71]. Unlike the other two isoforms of REG (REGα, REG β) that are predominantly localized in the cytoplasm, REGγ is mainly located in the nucleus and related to the 20S proteasome [72]. REGγ can interact with MYC. The C-terminal domain of MYC is responsible for this interaction between REGγ and MYC. Ectopic expression of REGγ suppresses MYC transcriptional activity and promotes the degradation of MYC. This study showed that the knockdown of REGγ significantly elevates the stability of the MYC protein. REGγ also negatively regulates MYC-mediated gene expression and cell growth [71].

Pirh2 (p53-induced RING-H2 protein), also called Rchy1, has an important role in tumorigenesis with ubiquitin ligase activity [73]. As shown in the studies of Hakem et al., Pirh2 can control MYC stability through polyubiquitination and proteolysis of MYC. Of note, Skp2 interacts with MBII and C-terminal domain of MYC and N- and C-terminal domain of Pirh2. It was also shown that in Pirh2-knocked down human RKO cells and Pirh2 deficient murine cells, the level of MYC protein significantly increased. This shows that Fbw7, Skp2 and Pirh2 play a critical role in MYC turnover [74].

The transcription factor PLZF (promyelocytic leukemia zinc finger), also known as zbtb16, belongs to the POZ-Krüppel (POK) family that binds to a specific DNA sequence and regulate various biological process including cell proliferation, differentiation, and organ development [75]. Wild type (wt) PLZF directly binds to the MYC promoter, which mediates repression of the MYC promoter and reduces the level of MYC mRNA and phosphorylation [76]. PLZF can regulate MYC post-transcriptionally, through its impact on the Akt\MAPK pathway. Indeed, PLZF modulates the MAPK pathway, decreasing phosphorylation of MYC at Ser-62. As well, it reduces phosphorylation of Thr-58, resulting in increased MYC stability whereas reducing its transcriptional activity [77].

In recent decades, the importance of microRNAs (miRNAs) as oncogene\tumor suppressors has been recognized. miRNAs are short non-coding RNA molecules ranging from 21 to 25 nucleotides in length, which bind to a target sequence within the untranslated region (3'-UTR) of an mRNA [78, 79]. miRNAs can regulate gene expression in a post-transcriptional manner. miR-34c is a member of the miR-34 family that targets MYC during DNA damage. To restrict Myc-induced DNA synthesis, repression of MYC by miR-34c is a crucial event in response to DNA damage. It inhibits continuous DNA synthesis and proliferation in the face of damaged DNA [80, 81].

## The role of MYC in cell cycle regulation

**Fig. 3:**
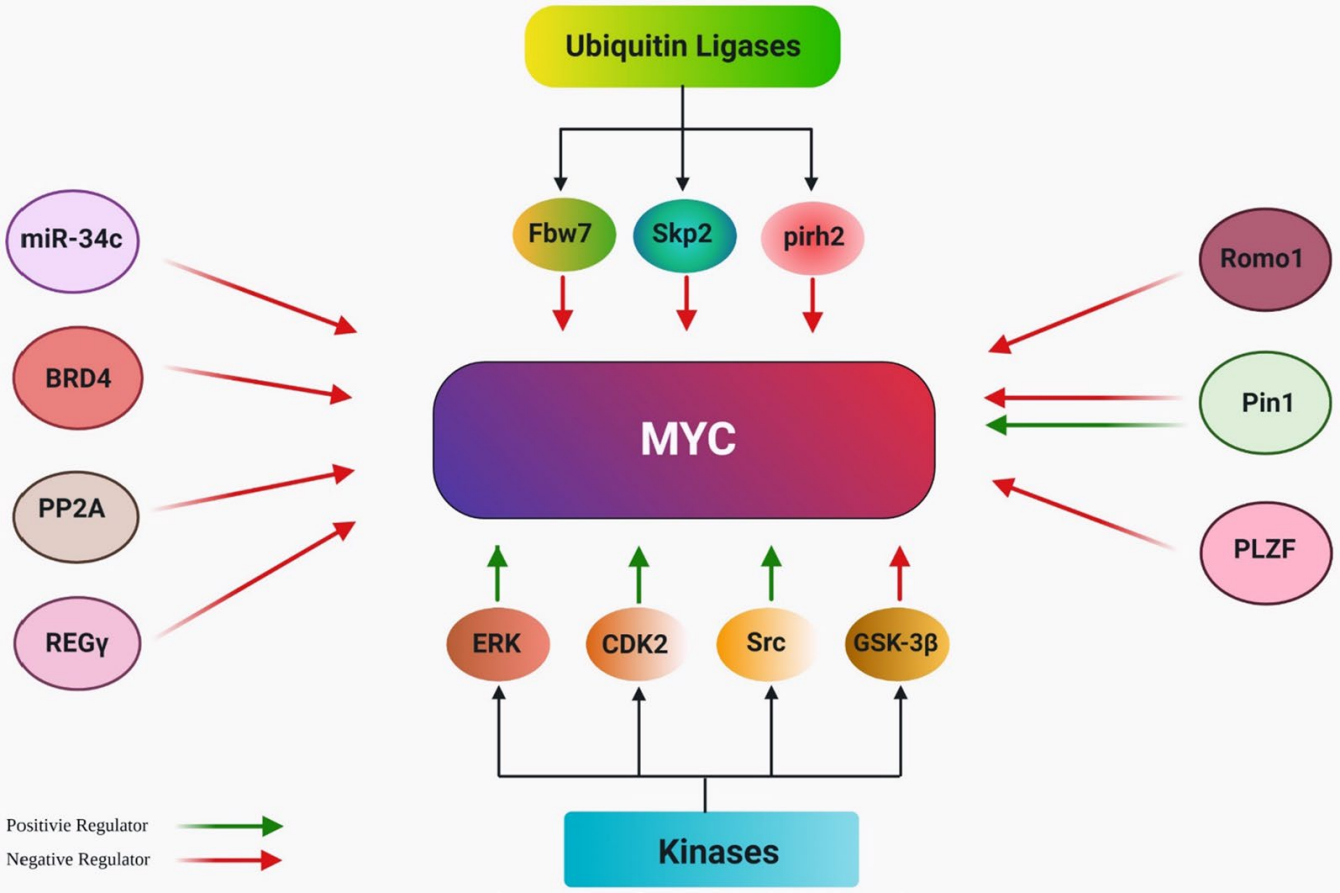
A brief overview of MYC regulation. Multiple regulators from different classes are involved in MYC regulation. Red and green arrows point to the negative and positive regulatory effects of each factor on MYC, respectively

**Fig. 4:**
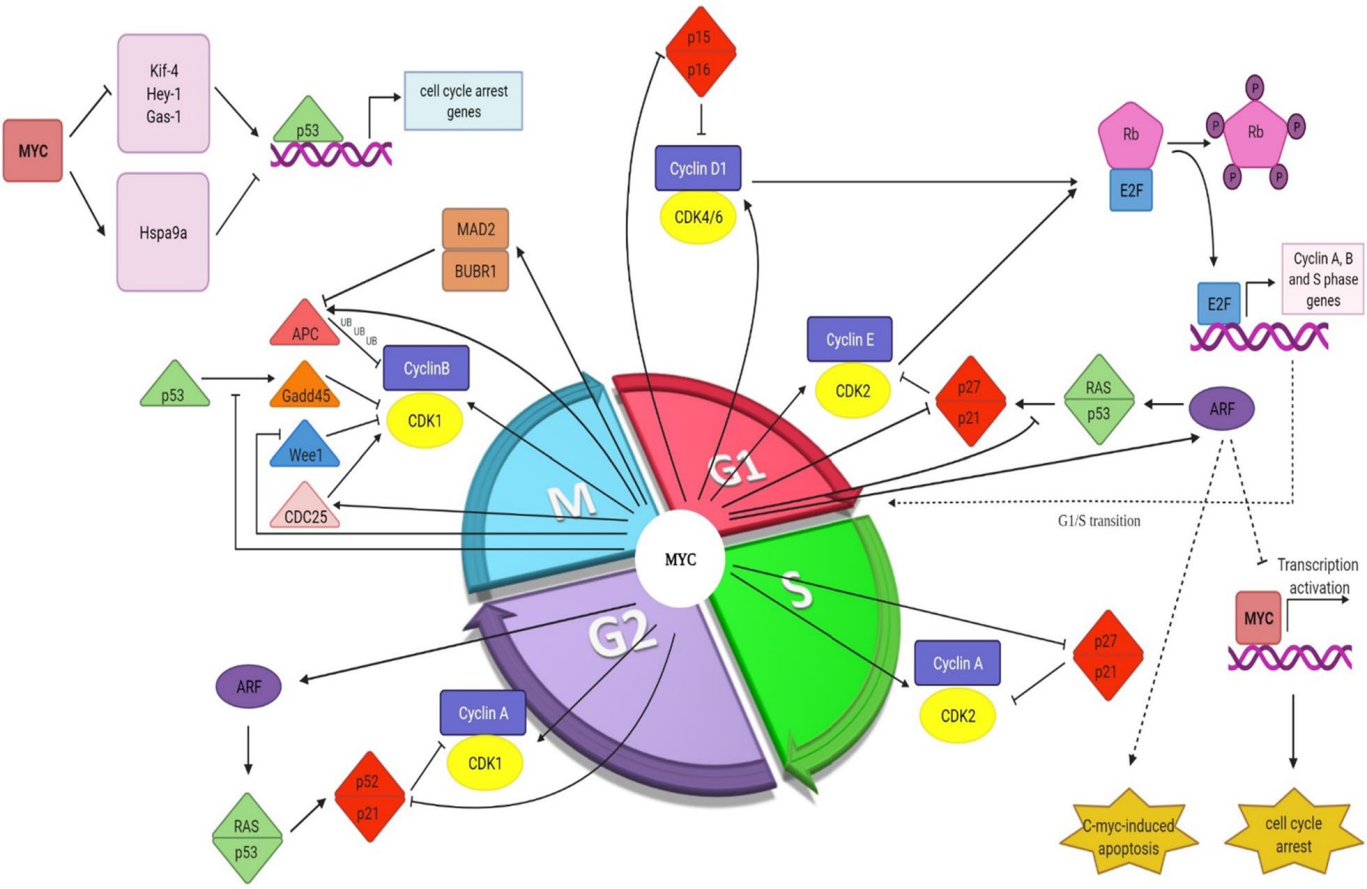
Diverse mechanisms are considered for MYC collaboration in cell cycle progression. The positive regulators of cell cycle are induced or activated by MYC. Through multiple mechanism, MYC blocks the activity of cell cycle regulators

The pivotal role of MYC in the cell cycle regulation and the proliferation rate has been assessed in several studies (Figs. 3, 4) [8–10]. Entering and exiting cell cycle in quiescent cells is achievable by MYC regulation [11, 12]. MYC has an important role in entering the G1 phase. This phase is longer in MYC deficient rat fibroblasts in comparison to the wildtype-cells [82]. The human ubiquitin ligase HectH9 contributes in MYC-mediated cell cycle progression and activation of target genes. In human tumor cell lines lacking HectH9, cells cannot progress beyond the G1 phase of cell cycle [58]. The stability of MYC is regulated by the Raf-MEK-ERK and the PI3K-Akt cascades. ERK-mediated MYC phosphorylation at Ser26 protects it from degradation, while GSK-3β phosphorylates MYC at Thr58 and exposes it to ubiquitin–proteasome mediated degradation. In the early G1 phase, Ras-induced ERK activation leads to GSK-3β inhibition, but in the end of G1 phase, GSK-3β is activated upon decreased function of Ras-PI3K-Akt pathway [40, 83, 84].

Ectopic MYC expression induces cells to enter S-phase and mediates mitosis in the absence of growth factors [85]. Schuhmacher et al. studied the effects of fine-tuned MYC protein on proliferation rate and cell cycle distribution in human lymphoblastoid p493-6 cell line. They observed that the steady increase in the fraction of cells in the S- and G2/M- phase, increased proliferation rate, and cell size relies on high levels of MYC in a dose-dependent manner, [86, 87]. Using MYC antisense oligonucleotides in human lymphoid and myeloid cells prevents entry into S-phase [88, 89]. Depletion of MYC in 23 cell lines with short-hairpin in a systematic study led to cell cycle arrest in G0/G1 in normal and some tumor-derived cell lines, whereas G2/M arrest happened in other tumor-derived cell lines [90]. Cell proliferation is hampered by the action of Mxd proteins that antagonize MYC transcriptional activity [37, 91, 92]. Expression of MadMYC a dominant negative MYC mutant containing the DNA binding and dimerization domains of MYC and the trans-repression domain of Mxd1 (also called Mad1), can inhibit the cell cycle arrest [93, 94]. The expression of this mutant blocks CCNB1 (cyclin B1) upregulation following stimulation of starved cells with serum [95]. In response to moderate hypoxia, HIF-1α inhibits MYC causing cell cycle arrest, but HIF-2α can reverse it [96, 97]. The results of these studies reveal that MYC regulates cell cycle progression.

A family of serine-threonine protein kinases consisting of a regulatory subunit, cyclin, and a catalytic subunit or CDK [98, 99]. The levels of cyclin oscillate throughout the cell cycle, resulting in CDK activation. In contrast, the activity of CDKs remains stable during the cell cycle (CDK1, 2, 4, and CDK6 for G1 phase, CDK2 for S phase, and CDK1 for G2 and M phases) [100]. Decreased activity of CDK4, 6 and CDK2 as well as prolonged G1- and G2-phase were seen in MYC-deficient cells [101]. Hypophosphorylated Rb sequesters and forms a complex with E2F1 transcription factor to suppress transcription of genes related to S-phase [102, 103]. At the early G1 phase, Rb is phosphorylated and E2F transcription factors are released upon the activation of CDK4/6 by D-type cyclins [104, 105]. G1/S transition relies on the CDK2 activation by cyclin E. Transition to S phase requires the expression of E2F target genes, which is dependent on further Rb phosphorylation by the action of cyclin E1/2-CDK2 in the end of G1 phase [106]. Cyclin E is degraded and replaced by cyclin A that is required for DNA replication and transition from S to M phase [107, 108]. CDK1 activation by B-type cyclins promotes transition into M phase [98, 109].

CDK inhibitory proteins (CKIs) comprising of the INK4 and the CIP/KIP (CDK interacting protein/kinase inhibitory protein) families downregulate CDKs and [110, 111]. The members of INK4 family including p15, p16, p18, and p19 bind to CDK4/6 to hamper their kinase activity and impair the CDK4/6-cyclin D interaction [110, 111]. p15 and p16 impede Rb phosphorylation and S-phase entry [112]. In response to high levels of p15, cell proliferation as the consequence of p27 redistribution from cyclin D-CDK4/6 to cyclin E-CDK2 is blocked [113].

ARF (alternative reading frame) gene is located in the INK4A/ARF/INK4B locus on chromosome 9p21 and shares the exon 2 and 3 with p16. ARF causes cell cycle arrest in G1 and G2 and favors MYC-mediated apoptosis via both p53- dependent and -independent pathways [114–116]. ARF sequesters MDM2 from p53, which is followed by p53 stabilization and activation and after that induction of p21 and other proteins triggers apoptosis in a p53-dependent pathway [117]. Moreover, ARF interaction with MYC has been shown in several studies [118, 119]. Following the elevation of MYC levels, ARF binds with MYC and prevents its transactivation ability to induce hyperproliferation and transformation; albeit, ARF cannot prevent MYC-induced apoptosis. The underlying mechanism is possibly due to the transrepression of particular anti-apoptotic genes by MYC [120–123].

The members of CIP/KIP family (p21, p27, and 57) are involved in the suppression of cyclinA, E, D/CDK complex catalytic activity [124–126]. MYC engages in cell cycle regulation not only by the upregulating of genes necessary for cell cycle progression but also by impairing the negative regulators of cell cycle [127, 128]. Different mechanisms are considered to explain MYC participation in cell cycle regulation. MYC- mediated E2F family induction by binding to E box in their promoter leads to S-phase progression [129–132]. In addition, RB phosphorylation by cyclin/CDK complexes rescues E2F transcription factors from inhibitory interaction with Rb and mediates the expression of E2F target genes implicated in cell cycle promotion [104, 105, 127]. The increased levels of cyclin/CDK complexes by MYC are mediated through different mechanisms, whether by inducing gene expression or by regulating phosphorylation and dephosphorylation of diverse residues of CDK proteins [128]. Moreover, MYC induces miR-221, which modulate Rb mRNA [133, 134].

It was demonstrated that MYC induces the CDK genes including CDK4 [135] and CDK6 [136, 137], but there is controversy with regards to CDK2. Yap et al. observed both mRNA and protein levels of CDK2 were induced upon MYC overexpression [137], but another experiment shows a different role for this gene [138]. Based on ChIP assay, MYC binds to CDK1 promoter [139], but other proteins such as Ras [140] or cyclin C [141] cooperate with MYC to augment the expression of CDK1. Increased activity of CAK (CDK activating kinase) by MYC is also required for complete activation of cyclin/CDK complexes, since CAK carries out the activating phosphorylation of CDK T loop [142–145]. Moreover, MYC counteracts the inhibitory phosphorylation of CDKs either by targeting Wee1 through miR-221 induction or provoking Cdc25 phosphatase [133, 134, 142, 143, 146, 147]. Mir-221 also targets mRNAs of p27, p57, and Rb [133, 148].

In addition to inducing CDK genes, MYC also regulates the expression of cyclins. MYC has conflicting roles in cyclin D1 regulation. Depending on the cell types, MYC can increase, suppress or not affect the expression of cyclin D1 [138, 149–152]. On the other hand, MYC induces the expression of cyclin D2 [153, 154], D3 [155], E1 [156, 157], E2 [157, 158], A [138, 159–161], and B1 [95, 162, 163]. MYC recruits TRRAP to induce histone acetylation and subsequently cyclin D2 expression [154]. MYC mediates cyclin E1 induction either directly or by inducing E2F transcription factors [164]. Serial analysis of gene expression was done by Menssen et al. to identify MYC target genes. CDC2-L1, cyclin B1, and cyclin E binding protein 1 are among the MYC-induced cell cycle regulators involved not only in G1/S transition but also in G2 progression [95].

Apart from inducing positive cell cycle regulators, MYC also represses the activity of cell cycle inhibitors [127]. TGF-β signaling inhibits MYC and induces p15 and p21 to mediate cell cycle arrest in G1 phase. TGF-β-induced p21 is abolished through AP4 transactivation by MYC [165]. TGF-β treatment in lung epithelial cells downregulates MYC rapidly and induces p15 expression. Exogenous MYC expression blocks TGF-β -induced p15 expression [166]. After TGF-β treatment, Miz-1 binds to transcriptional initiator site (Inr) within the proximal region of the p15 promoter to augment p15 activity [167, 168]. MYC collaborates with other proteins, including Miz-1, SP1, and SMAD to block p15 induction. MYC and SP1 switch from transcriptional activator to transcriptional repressor upon their interaction with MYC and following their co-activators replacement [167–170]. Miz-1-mediated p300 recruitment and p15 induction are at the mercy of Miz-1 interaction with MYC [167, 168]. MYC forms an inhibitory complex with SP1 and SMAD to repress p15 upon TGF-β treatment [170].

Induction of E2F1 transcription factor, which induces ARF expression, and counteracts ARF ubiquitination and degradation by ULF ubiquitin ligase has been considered for MYC-induced ARF upregulation [171–174].

MYC regulates p21 in different ways. It overrides p21 induction by p53 and paves the way for p53-induced apoptosis [175]. Cell cycle cessation upon DNA damage is thwarted by MYC counteraction with p53-induced p21 and GADD45 [176–180]. Nuclear localization of Cyclin B1 is reduced by the action of GADD45, yielding Cdc2 kinase activity inhibition [181]. The binding of MYC and Miz-1 is one of the mechanisms that either directly inhibits p21 expression or indirectly via recruiting the DNA methyltransferase DNMT3a [182–184]. The ability of MYC to form a ternary complex with histone demethylase KDM5B and the transcriptional factor TFAP2C conflicts with p21 induction [185]. Besides, MYC interference with SP1 and Ras-mediated p21 induction [186, 187], and MYC-induced transcription factor AP4 [188] and miR-17-92 [189, 190] result in p21 suppression.

The expression of p27 and MYC shows an opposite pattern in several studies, as high levels of MYC are associated with low levels of p27 [191–194]. MYC represses p27 at both transcriptional and post-transcriptional levels. p27 regulation at the transcriptional level is mediated by MYC interaction with Inr element within the p27 promoter or its interaction and inhibitory effect on Foxo3a, a transcription factor required for p27 upregulation [195, 196]. MYC-mediated mir-221 and miR-222 upregulation repress 27 at the post-transcriptional level [134, 148]. MYC-induced cyclin E transcription directly or through E2F transcriptional induction bypasses G1/S arrest and antagonizes p27 [156, 197, 198]. MYC sequesters p27 from cyclin E-CDK2 complex by inducing the expression of D-type cyclin and CDK4 and CDK6 [153, 199, 200]. MYC-mediated cyclin E induction also stimulates p27 phosphorylation at Thr-187 and makes it more prone to be recognized and degraded by the SCF^SKP2^ ubiquitin ligase complex. Moreover, the expression of several subunits of SCF^SKP2^ ubiquitin ligase complex are induced by MYC to promote p27 proteasome degradation [201–206].

The transcription factor FoxM1 is a MYC target gene and controls G2/M promotion [207]. MYC targets several genes related to DNA replication and mitosis. It promotes DNA replication in both transcriptional-dependent and -independent manner. A number of origin recognition complex (ORC) genes, including ORC1, ORC2, ORC4, and ORC5 are among MYC target genes [137, 157]. Ctd1 [208], the main component of the pre-replicative complex, cdc6 [136, 157] and MCM proteins [157, 209, 210] as MCM3, MCM4, MCM5, and MCM6, proteins required for initiation and elongation of DNA replication, are induced by MYC. MYC also interacts with pre-replicative complex to increase replication origin activity [211]. In addition to CDK1, MYC regulates other genes encoding proteins involved in mitosis. MYC mediates mitosis progression by provoking subunits of anaphase promoting complex/cyclosome (APC/C), including Anapc5, cdc16, and cdc23, to increase the degradation of cyclin B1 and securin, which controls the transition of metaphase-anaphase transition [137, 212, 213]. On the other hand, it was shown that MYC has a different role as Anapc2. As well as securin degradation, MYC represses securin gene expression (PTTG1) [137]. Cells overexpressing MYC showed delayed anaphase onset through transactivation of MAD2 (mitotic arrest deficient) and BubR1 [214].

A study reported that in response to anti-mitotic drugs, such as taxol, nocodazole, Eg5 inhibitor, and other drugs disrubpting mitosis, MYC augments apoptosis [215]. Following treatment with the aforementioned agents, cells with low levels of MYC showcased less apoptosis compared to cells having high levels of MYC. Besides, cells overexpressing MYC exhibited more anomalies, since MYC exacerbates drug-induced micronuclei formation, a hallmark of chromosome instability [215]. Although normal mitosis took place in both high- and low-levels of MYC, mitotic timing and spindle morphology were under the control of MYC levels. In cells having high level of MYC, the spindle length and metaphase plate width were reduced and increased, respectively. Furthermore, acceleration of nuclear envelope breakdown (NEBD) to metaphase and delayed anaphase was seen in cells with high level of MYC. MYC modulates mitosis by controlling mitosis related events, including centriole biogenesis, kinetochore assembly, proteolysis,abscission, and cytokinesis [215].

Ciribilli et al. identified the genetic events associated with cell cycle and apoptosis in MYC transgenic lung tumors [216]. The expression of CDK4 and its related cyclin D1, and transcription factor DP1, a heterodimeric partner of E2Fs, were increased, while p19 was downregulated. CDK1 and cyclin B1 and B2 were overexpressed. In addition, upregulation of several genes such as the serine/threonine kinases Nek6 and Stk6 (Aurora-A kinase), Cks1 (cdc28 protein kinase), Cks2 (cdc28 protein kinase regulator subunit 2), cdc20, regulators of cytokinesis Prk1 (protein 1) and kinesin family members were seen. Moreover, increased ect2 expression, an oncogene required for cytokinesis [217], and downregulation of Lats2 that is a negative regulator of cell cycle [218] were observed. Another transcriptional alteration in lung tumors of MYC transgenic mice models is reversal of p53-induced cell cycle arrest mediated by repression of transcription factors involved in regulation of p53 activity. These include Klf-4 [219], Hey-1 [220], Gas-1 [221], and Hspa9a [222].

In addition to inducing miRNAs that target the negative regulators of cell cycle, MYC also suppresses miRNAs that acting as barriers to cell cycle progression [223]. Let-7 family members, miR-15a/16-1, miR-26a, and miR-34a are among the targets of MYC. Let-7 miRNAs regulate Cdc25a, CDK6, cyclin A, cyclin D1, D2, and D3. MiR-34a negatively regulates expression of CDK4, CDK6, cyclin E2, and E2Fs; miR-15a/16-1 participates in the regulation of CDK6, E2F3, cyclin D1 and D3; and miR-26a represses cyclin D2 and E2 [224–227].

MYC also induces the H19 long-non coding RNA (lncRNA), which silences Rb and promotes proliferation [228–231]. MYC-induced long noncoding RNA (MINCR) is another LncRNA induced by MYC that has a close correlation with its expression. MYC binding to the promoter of selective cell cycle genes is weakened following MINCR knockdown [232].

## The role of MYC in DNA damage response

**Fig. 5:**
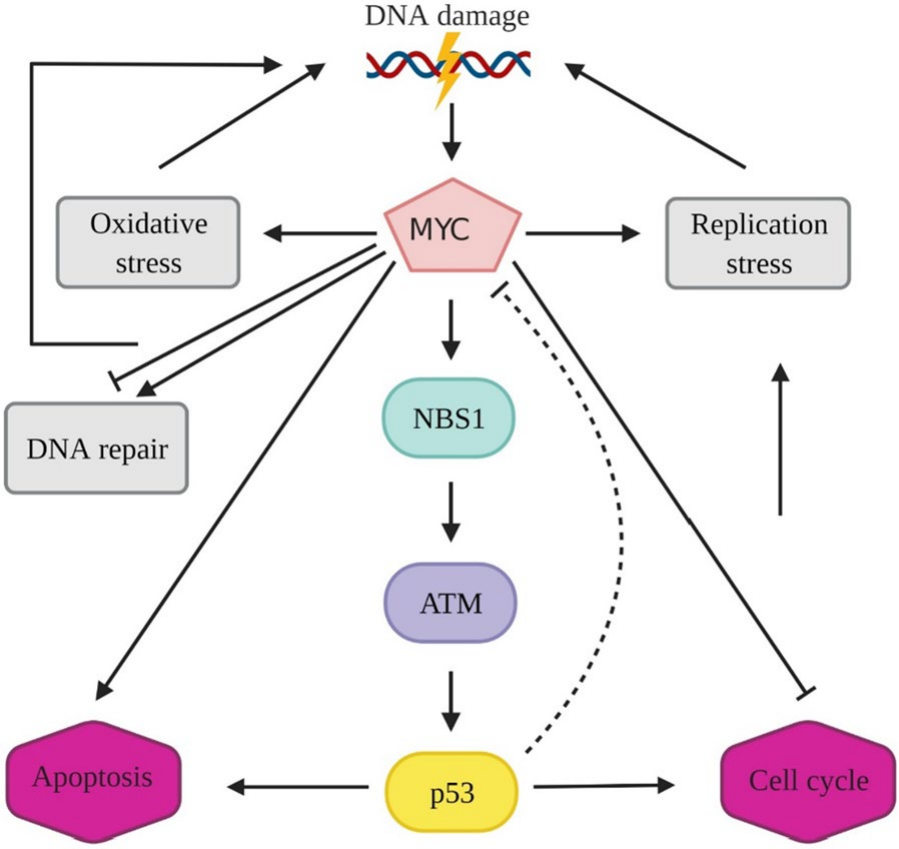
MYC has multiple effects on the DNA damage response. MYC is essential for NBS1 expression and the activity of ATM and its downstream effectors such as p53. In turn, p53 suppresses MYC expression in a pulsatile pattern. MYC drives cell fate toward apoptosis and overrides cell cycle arrest. MYC also participates in the generation of DNA damage. DNA repair impairment, ROS generation, and increased replication stress are among other MYC-provoked DNA damage response mechanisms

The role of MYC in DNA damage signaling has been investigated in numerous studies. MYC levels are decreased through different mechanisms depending on the extent of DNA damage, including alternation in MYC transcription and protein turnover [14–16]. The results of several studies exhibit that decreased levels of MYC are assumed as a step in DDR [15, 17, 18] (Fig. 5).

Decreased MYC protein levels and p53 accumulation has been observed in UV-induced DNA damage in which the proteasome-dependent degradation mechanism was implicated for MYC level reduction [14]. Jiang et al. suggested tripchlorolide-induced DNA damage causes proteasome-dependent MYC degradation, resulting in apoptosis induction [15]. Moreover, Fbw7 ubiquitin ligase mediates MYC degradation in response to DNA damage due to the USP28 disassociation from Fbw7 [18], which is required for MYC stability [233]. Exposure of MCF-7 breast tumor cell line to DNA-damaging agents such as topoisomerase II inhibitors VM-26, m-AMSA, and doxorubicin, or ionizing radiation is capable of suppressing MYC mRNA [234–237]. Moreover, continuous treatment of MCF-7 with VM-26 suppresses both mRNA and protein levels of MYC, and its transcriptional activity in response to sustained DNA damage [238]. Treatment of MCF-7 cells with camptothecin at the concentrations, causing DNA damage results in MYC suppression, while in lower concentrations, this attenuation in MYC expression vanishes due to the absence of detectable DNA damage [239]. In response to DNA damage, pRb is dephosphorylated [240] and in the complex with E2F1 represses MYC. Another factor influencing MYC is a acetyl-transferase called TIP60, which has a co-regulator activity towards MYC. Intriguing, TIP60 obstacles tumor progression by modulating DDR [241], and its low levels in different cancers are associated with tumor progression and inferior survival [242].

MYC-mediated activation or repression of many target genes such as Bax, GADD45A, and ONZIN are involved in DDR [243–245]. Pusapati et al. observed MYC overexpression in a transgenic mouse model causing p53 activation following DNA damage and ATM was required for p53 activation to augment apoptosis and interfere with MYC-mediated tumorigenesis [246]. MYC exists upstream of PI3K related to DDR and augments signal transduction following DNA damage [247]. This oncogene has multiple effects on DDR.

MYC can enhance DDR, as the activation of ATM-dependent checkpoints relies on it. Guerra et al. observed that in response to DNA damage, nuclear foci formation of the Nijmegen breakage syndrome 1 (Nbs1) and subsequently phosphorylation and activity of ATM and its downstream effectors were reduced in the cell line lacking MYC, resulting in impairment in p53stabilization and delayed DDR [248]. A previous study showed Nbs, a member of MRN complex, is a target gene for MYC [249]. Nbs1 senses DNA breaks and is essential for ATM activation in the presence of DNA damage [250, 251]. Therefore, MYC-mediated Nbs1 expression affects DNA damage-induced signal transduction pathways. In unstressed conditions, Miz1 associates with topoisomerase II binding protein1 (TopBP1), but upon UV irradiation, TopBP1 detaches from Miz1 [19]. TopBP1-Miz1 association negates TopBP1 proteasomal degradation that ATR-dependent signal transduction is relied on. MYC has a negative effect on ATR-dependent signal transduction in response to DNA damage. This involves TopBP1-Miz1 disassociation and TopBP1 degradation by HectH9 [252].

Upon DNA damage, p53 induces the expression of p21 [176] and GADD45 [177] to mediate cell cycle arrest. MYC has the opposite role, and it represses these genes [178–180] and attenuates p53-mediated cell cycle arrest [253, 254]. However, it drives p53 functions toward apoptosis induction instead of cell cycle arrest [255]. Upon DNA damage induced by gamma irradiation and daunorubicin, MYC interacts with Miz-1 and downregulates p21 expression to favor apoptotic arm of p53 signaling [255]. MYC forms a transcriptional repressor complex with Miz-1 in order to suppress p21 [19, 126]. Transactivation of AP4 by MYC allows cells to re-enter cell cycle even in the presence of DNA damage. Jung et al. showed that after treatment of MCF-7 cells with etoposide, the protein levels of MYC and AP4 were reduced. To the contrary, p21 and p53 levels were elevated. These results show AP4 abrogates p53-mediated cell cycle arrest by suppressing p21 and favors apoptosis induction, resulting in cells sensitization to DNA-damaging agents [165].

MYC can inhibit both G1/S and G2/M arrest. After irradiation, in human mammary epithelial cells (HMEC) overexpressing MYC, G1/S arrest was impaired due to inappropriate hyperphosphorylation of Rb and subsequent reappearance of cyclin A, which leads to the entry of cells into S phase [256]. Overexpression of MYC also attenuates G2/M arrest by upregulating cyclin B1, and stimulates cells with damaged DNA to enter mitosis [257]. The human ubiquitin ligase HectH9 is essential for MYC-mediated cell cycle progression and activation of target genes. In human tumor cell lines lacking HectH9, cells accumulate in the G1 phase of cell cycle [58]. In the study by Robinson et al. primary human fibroblasts overexpressing MYC exhibited accelerated S phase in contrast to prolonged S phase in the cells lacking MYC. They showed the Werner DNA helicase protein (WRN) is required for the repair of stress-induced DNA damage. WRN depletion resulted in DNA damage accumulation in cells overexpressing MYC [258].

MYC promotes apoptosis by bypassing p53-mediated cell cycle arrest [19, 126, 255] and drives cells with damaged DNA toward S phase [247, 259]. Moreover, MYC suppresses the expression of anti-apoptotic proteins such as BCL-XL and BCL-2 [260]. PER1 can push DDR in favour of apoptosis via upregulating MYC. In response to DNA damage, high levels of MYC and concomitantly p21 reduction were seen in cells overexpressing PER1, highlighting the positive effect of PER1 on apoptosis induction [261]. On the other hand, heat shock protein HSP70 opposes MYC-evoked apoptosis in response to etoposide and camptothecin [262]. BRCA1 stands in the way of apoptosis induction ensuing exposure to DNA damaging agents [263, 264]. Further investigation revealed that BRCA1 cooperates with MYC to suppress psoriasin, resulting in resistance to etoposide [265]. Upon treatment with DNA damaging cytotoxic drugs, the physiological level of MYC is required for strong apoptosis induction through the activation of Bid and Bax and pro-apoptotic enzyme PLKδ. Apoptosis is abrogated in MYC null cells, confirming that apoptosis is dependent on expression of this oncogene [266, 267].

MYC depletion in colorectal cancer cell lines promotes cell-cycle arrest rather than apoptosis due to the alternation in p53 signaling and its downstream effectors. In fact, p21 is increased, whereas the levels of pro-apoptotic genes such as Bax are decreased [255]. Moreover, under irradiation, fibroblasts undergo apoptosis as a result of MYC function that targets BCL-XL [268]. In colon cancer cell lines overexpressing MYC, camptothecin treatment results in effective apoptosis induction, indicating MYC overexpression contributes to colon cancer cells sensitization to this agent. Following camptothecin treatment, the levels of p53 and its target genes are upregulated, while overexpression of MYC induces p53 and overrides p21 induction [269].

The mechanism underlying chemosensitivity in small cell lung cancer cell lines harboring p53mutations was investigated by Supino and colleagues [270]. They showed upon treatment with doxorubicin, MYC is upregulated to induce apoptosis independent of p53 and renders the cells more prone to chemotherapy. The Y box binding protein (YB1) is a transcription factor with the ability to modulate the outcome of anticancer agents treatment and is responsible for drug resistance [271–273]. p73 interacts with MYC and promotes the formation of MYC-MAX complex to increase the transcriptional activity of MYC, resulting in YB1 upregulation-mediated drug resistance [274].

Etoposide causes DNA damage in S phase and provokes apoptosis in G2 [275]. A study showed MYC is indispensable for apoptosis in G2 phase. The same does not hold true in G1 phase [276]. It was observed that treatment of MYC null cells with etoposide abrogated apoptosis, while cisplatin exposure causes DNA damage irrespective of cell cycle stage.

MYC is capable of activating the transcription of genes involved in DNA repair, including NBS1, KU70, BRCA2, Rad50, and Rad51 [136, 210, 249, 277]. Cui et al. demonstrated that MYC affects the repair of DSB (double strand breaks) caused by ionizing irradiation (IR). The ability to repair DSB was attenuated in MYC-knockdown cells Hela-630 after exposure to IR due to the reduction in DNA damage-induced ATM phosphorylation and DNA-PK kinase activity [278]. Moreover, the upregulation of genes involved in the repair of DSBs through HR and NHEJ is dependent on MYC and HIF2α [279].

It has been suggested that MYC participates in p53 regulation [280–282]. Phesse et al. observed DNA-damaging agents can no longer cause apoptosis when MYC is deleted in the adult murine cells [282]. Tight regulation of MYC levels is essential for precise kinetic apoptosis in response to DNA damage. Treatment of Rat-1 fibroblast cell line with DNA-damaging agent VP-16 demonstrated MYC is required to achieve the optimal apoptotic response [283].

It has been suggested that p53 is involved in MYC modulation. Besides the transactivation of genes involved in cell cycle arrest, p53also represses MYC through a mechanism dependent on histone deacetylation [284]. Following irradiation, the mRNA levels of MYC were reduced in AML-3 cells expressing wild type p53. In K562 cells lacking p53however, there was no reduction [285]. In some studies, the dynamic behavior of p53accounts for its broad function. Several models have been proposed for acquiring p53dynamic response [286–288]. Porter and colleagues showed that MYC and p53have an inverse concentration relationship [289]. In response to DSB, p53induces MYC repression in a pulsatile pattern, which is thought to be due to p53binding to downstream enhancer within a MYC super-enhancer region [290]. This is consistent with previous studies that showed p53 has the potential to suppress MYC [291, 292]. A study stated that MYC, a non-linear transcription factor is capable of universally affecting active genes, but not ones induced priorly to MYC [6, 293]. p53-mediated MYC repression however has different impacts on global transcription. Cell fate is affected by this transcriptional inhibition effect of p53 on MYC. Proper cell cycle arrest and apoptosis prevention are the consequence of p53-mediated MYC repression.

Several miRNAs are involved in the negative regulation of MYC [294–296]. In a study that was done by Cannell and colleagues [80], the levels of p53, p21, and miR-34c were upregulated in HEK293 after treatment with etoposide, while MYC protein levels but not MYC mRNA levels were decreased. p53is a crucial regulator of miR-34 family. These are important mediators of DDR [297–301] that control MYC levels [302, 303]. Nevertheless, it has been shown that p38 MAPK/MK2 pathway also mediates miR-34c induction to prevent aberrant MYC-induced replication even in the absence of p53 [80]. In fact, DNA damage-mediated miR-34c induction gives rise to MYC repression to halt cell cycle at the S phase and counteract DNA synthesis and replication. Li et al. showed that after exposure to UV-induced DNA damage, ribosomal protein L11 is released from the nucleolus to the cytoplasm to promote miR-130a recruitment, resulting in decreased levels of MYC mRNA and protein [304].

Upon DNA damage, bridging integrator 1 (BIN1), a nucleocytoplasmic protein, is activated to mediate apoptosis [305, 306]. Loss of BIN1 attenuates cell response to DNA-damaging agents [306]. This adaptor protein interacts with MYC and perturbs MYC-mediated transactivation of target genes [305, 307, 308]. Pyndiah et al. observed that regardless of TP53 gene status, BIN1 exerts essential roles in enhancing DNA damage caused by cisplatin. The mechanism behind this chemosensitivity is dependent somewhat on BIN1-MYC interaction. BIN1 interaction with PARP1 which is followed by inhibition of the latter is another mechanism that inhibits MYC-induced transactivation, G2/M transition, and sensitizing cells to DNA damage [309]. PARP1 acts as a scaffold, and interacts with proteins involved in the base excision repair (BER) [310–312]. BIN1-mediated PARP1 inhibition also impairs BER pathway and results in chromosomal destabilization. Moreover, BIN1 inhibits indoleamine 2,3-dioxygenase (IDO), resulting in the intracellular NAD reduction and PARP1 activity restriction [313–315]. In addition to PARP1, BIN1 interacts with proteins involved in non-homologous end joining (NHEJ) pathway [316, 317]. It is noteworthy that MYC overexpression restores PARP1 activity by blocking BIN1 activation by Miz-1 to overcome BIN1-mediated PARP1 repression [309].

In another study, the regulatory effects of epigenetic alterations on DDR have been demonstrated [318]. SMAD nuclear interacting protein 1 (SNIP1) has the potential to regulate DDR, apoptosis, and cell cycle [319, 320]. SINP1 interacts with the ten-eleven translocation dioxygenase 2 (TET2). This interaction mediates TET2 binding to several transcription factors such as MYC in order to recruit TET2 to the promoter of MYC target genes and therefore regulates their expression [318].

In addition to being involved in DDR modulation, MYC is also considered an element that causes genomic instability [253, 259, 321–325]. Several mechanisms contribute to MYC-elicited genomic instability. These include inducing DNA damage, gross chromosomal rearrangement, aberrant cell cycle progression, and disrupting DNA repair processes [326]. Moreover, cells with damaged DNA like MYC-induced DSB can re-enter the cell cycle in response to MYC overexpression, resulting in genomic instability [253]. In addition, MYC can promote DNA damage independent of ROS [324]. Increased DNA replication stress is another mechanism that underlies DNA damage induced by MYC [323, 327–329]. Dominguez-Sola et al. noticed the non-transcriptional role for MYC in DNA replication. This oncogene interacts with pre-replicative complex and modulates replication origin activity. They showed MYC overexpression increases replication origin activity, and consequently persuades replication-dependent DNA damage [211]. In addition to non-transcriptional control of DNA replication, MYC activates CTD1 transcription [208], a key component of pre-replicative complex required for origin licensing [330–332]. It was shown Polη, a Y-family translesion synthesis polymerase, relieves MYC-induced replication stress by mediating fork progression to suppress DSB formation [333]. P300 regulates MYC negatively and so counteracts aberrant DNA synthesis [334, 335]. P300 knockdown results in entry into S phase followed by deregulated replication origin activity and DNA synthesis due to MYC induction, leading to the DDR-activated apoptosis [336].

MYC can also play a role in genomic instability through interrupting DSB repair. It has been observed that MYC has the potential to interfere with DNA damage repair [259, 337]. Li et al. showed MYC inhibits the repair of DSBs caused by IR or the action of RAG1/RAG2 during V(D)J recombination [338]. Based on ChIP array studies, there is an association between MYC and the promoter of several DSB repair-related genes including Rad51, Rad51B, Rad51C, XRCC2, Rad50, BRCA1, BRCA2, DNA-PKcs, XRCC4, Ku70, and DNA ligase IV [136, 210, 249, 339]. Song et al. showed that MYC disrupts homologous recombination-mediated DNA repair through upregulating miR-1245 to suppress BRCA1 expression, resulting in hypersensitivity to γ-irradiation [337]. In tyrosine kinase-activated leukemias, elevated generation of ROS, and DNA damage as well as activation of error-prone repair process has been seen [340, 341]. Muvarak et al. [342] proposed MYC overexpression is involved in the activation of alternative NHEJ, an error-prone repair pathway [343, 344], via upregulating the expression of LIG3 and PARP1, which is dependent on expression of FMS-like tyrosine kinase-3 (FLT3)/ITD and BCR-ABL1. Jin et al. showed BCL2 binds to MYC and boosts its transcriptional activity to hindering DNA repair through targeting APE1, a member of the BER pathway [345].

Partlin et al. observed that MYC has the potential to interact with MLH1 mismatch repair protein and inhibits its activity [346]. It was reported that MYC downregulation rendered melanoma cells susceptible to IR-induced apoptosis through inhibition of MLH1 and MSH2, resulting in DNA repair prevention and DNA damage accumulation, followed by induction of apoptosis in a p53-independent manner [347].

## MYC and apoptosis

A well-known fundamental function of MYC is the property to sensitize cells to apoptosis. The first oncogene reported inducing apoptosis was MYC [20]. Deregulated MYC expression, along with anti-proliferative signals, can lead to apoptosis [348]. Even in the presence of survival factors, deregulated MYC sensitizes cells to apoptotic stimuli such as irradiation, hypoxia, heat shock, interferons, TNF alpha, and Fas [349]. MYC needs to bind to DNA with its partner Max to induce apoptosis [20].

Two main pathways initiate apoptosis, the intrinsic (mitochondrial) and extrinsic pathways. These two pathways have a pivotal role in MYC-induced apoptosis. Different cellular stresses such as oncogene activation, DNA damage, and hypoxia can initiate intrinsic pathway and release of apoptogenic factors, including cytochrome c (cyt c), smac\DIABLO, and apoptosis-inducing factor (AIF) [350]. The release of mitochondrial cyt c into the cytosol facilitates the formation of the apoptosome complex, consisting of cyt c, Apaf-1, and procaspase-9. The apoptosome complex activates caspase-9 to directly cleave and activate effector caspases, caspase-3, and caspase-7. These caspases finally trigger apoptosis [351]. BCL-2 protein family has an essential role in regulating mitochondrial-outer-membrane permeabilization (MOMP), which is required for the release of cyt c. BCL-2 family has three subfamilies based on their functions: (1) anti-apoptotic members (BCL-2, BCL-X_L_, MCL-1, etc.), (2) BH3-only (pro-apoptotic) proteins (BAD, BID, BIK, BIM, PUMA, NOXA, etc.), (3) pore-formers or ‘executioner’ (pro-apoptotic) proteins (Bax, Bak, Bok) (9). Bax and Bak induce the release of cyt c by oligomerization and forming pores in the mitochondrial outer membrane. Anti-apoptotic proteins such as BCL-2 and BCL-X_L_ inhibit Bax and Bak translocation and oligomerization, resulting in suppression of MOMP and prevention of cyt c release [352, 353]. The balance between pro- and anti-apoptotic members regulates the release of cyt c from mitochondria so that when anti-apoptotic proteins are predominant, they inhibit the release of cyt c [354].

On the other hand, the extrinsic pathway is initiated through death-ligand binding to cell-surface death receptors. Death receptors are a subset of the tumor necrosis factor receptor (TNFR) superfamily that contains eight members: TNFR1, Fas (CD95), DR3, TNF-related apoptosis-inducing ligand receptor 1 (TRAILR1; also called DR4), TRAILR2 (DR5), DR6, ectodysplasin A receptor (EDAR) and nerve growth factor receptor (NGFR). The presence of about 80 amino acid death domain (DD) in the cytoplasmic region of death receptors has an essential role in activating the signaling cascade and induction of apoptosis [355]. Death receptors ligation create a death-inducing signaling complex (DISC), including adaptor molecule FADD, the initiator procaspase-8\10, and an inactive homolog of caspase-8, c-FLIP (cellular FLICE-like inhibitory protein). Interaction between FADD with procaspase-8 promotes homodimerization and autocatalytic cleavage of procaspase-8, leading to the formation of active caspase-8. This active form in turn cleaves and activates downstream caspases such as caspase 3 and 7 that execute cell apoptosis [356–358]. Caspase-8 also cleaves Bid pro-apoptotic protein to truncated Bid (tBid), which can translocate to mitochondria and release cyt c by inducing MOMP. Bid acts as a bridge between extrinsic and intrinsic apoptosis pathways [359, 360]. c-FLIP is a master anti-apoptotic regulator for death receptor-mediating apoptosis, which carries out its function by competing with procaspase-8 to bind to FADD, thus interferes with caspase-8\FADD interaction [361].

**Fig. 6:**
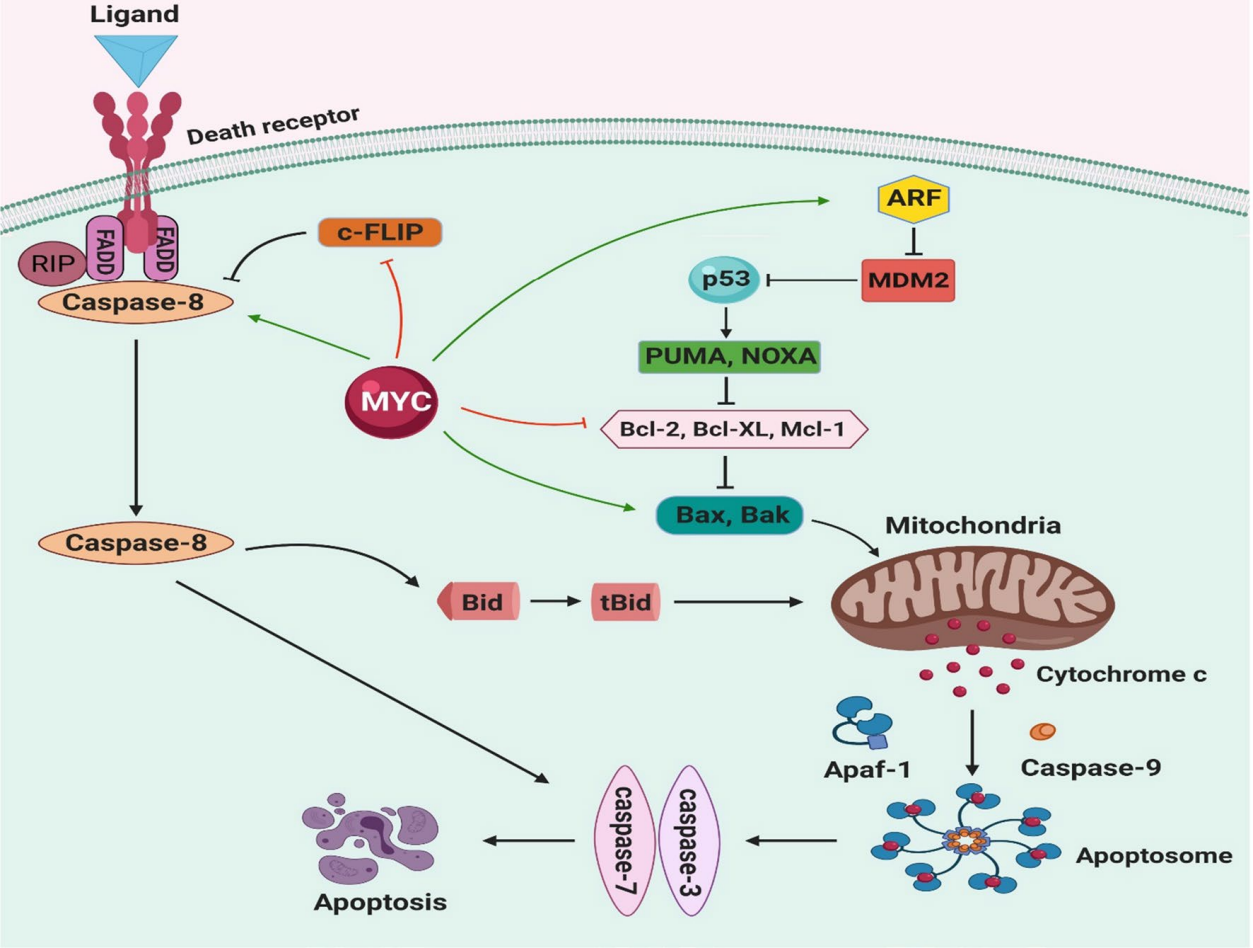
MYC and apoptotic pathway. Expression of MYC can sensitize cells to a broad range of proapoptotic stimuli such as DNA damage, death receptor, hypoxia, and nutrition deprivation. Through various pathways and possibly by inducing activation of Bax proapoptotic molecule, MYC promote the release of cytochrome c from mitochondria into the cytosol. Activation of Bax forming pores results in mitochondrial outer membrane permeabilization (MOMP). When cytochrome c releases into the cytoplasm, it interacts with APAF-1 and procaspase 9 to form apoptosome. Caspase 9 is activated in the presence of ATP, which in turn cleaves and activates caspase3 and 7, eventually triggering apoptosis. MYC is also involved in the death receptor pathway of apoptosis. Ligand-death receptor binding initiates interaction of adaptor molecules like FADD with death receptor. FADD auto-activates by recruiting procaspase 8. Caspase 8 can directly activate caspase3 and 7. Caspase 8 can also activate BH3-only protein BID, which stimulates MOMP. MYC induces apoptosis by p53dependent and independent mechanisms. Regulation of p53\MDM2\ARF by MYC, can stabilize p53and promote apoptosis

When studies show that MYC could provoke translocation of cytochrome c from the mitochondria, it was suggested that MYC could play a role in apoptosis [362]. The activation of Bax and Bak by MYC is an upstream mechanism of cytochrome c release (Fig. 6). Bax is one of the transcriptional targets of MYC and the primary mediator of MYC-dependent apoptosis. Expression of MYC induces Bax upregulation or, indirectly, controls Bax oligomerization [363, 364]. Bax and Bak are required for efficient apoptosis response, and MYC activation alone is not adequate to provoke apoptosis; hence Bax and Bak's deficient cells are significantly resistant to MYC-induced apoptosis [365]. Overexpression of BCL-X_L_ acts as a barrier that inhibits the MYC-induced conformational activation of Bak. Indeed, BCL-X_L_ is a pivotal factor for the mitochondrial apoptosis pathway by its inhibitory effect on Bak activation [366]. Recent studies indicate MYC can induce suppression of both BCL-2 and BCL-X_L_ anti-apoptotic proteins. By blocking MOMP through Bax and Bak inhibition, MYC-dependent apoptosis is prevented [367]. Based on the evidence provided by Muthalagu et al., Bim pro-apoptotic protein is a major mediator of MYC-dependent apoptosis in several solid tissues. MYC appears to stimulate apoptosis through binding to the Bim promoter and elevates Bim expression [22].

MYC is known as a stimulant that can sensitize cells to several death stimuli such as TNF-α, CD95/Fas, and TRAIL [368, 369]. The molecular mechanism that MYC promotes extrinsic apoptosis pathway is not well established; however, the inhibitory effect of MYC on the NF-kB pathway and suppression of survival genes along with its pro-apoptotic activities has been proposed [370–372]. Klefstrom and colleagues showed that receptor-interactive protein (RIP) is a serine\threonine kinase that initiates programmed cell death by.

FADD and caspase-8 dependent pathway. The MYC-mediated promoted expression of RIP can significantly enhance the apoptotic activity via FADD, caspase-8. Both caspase-8 and FADD are crucial for apoptotic synergy between RIP and MYC. [373]. MYC also acts as an inhibitor of c-FLIP expression, which enhances TRAIL-dependent activation of caspase-8 and apoptosis. The ectopic expression of FLIP represents the suppression of MYC-induced apoptosis. Caspase-8 can be increased directly or indirectly through post-translational modification by MYC [374]. Previous studies demonstrated that MYC also induces FasL up-regulation in T-lymphocytes and increases susceptibility to Fas-mediated apoptosis [375].

p53 pathway can become involved in MYC-dependent apoptosis through several mechanisms. p53 accumulates in the nucleus, where it is activated, and promotes growth arrest and/or apoptosis. It stimulates multiple apoptotic genes that have an important role in a different stage of apoptosis by transcription-dependent and independent mechanisms. p53 induces apoptosis via both intrinsic and extrinsic pathways [376, 377]. Post-translational modifications such as phosphorylation, acetylation, ubiquitination, and methylation as well as protein–protein interactions with cooperating factors stabilize and activate p53 [378]. Stable p53 can interact with pro-apoptotic genes such as Puma, Noxa, Apaf-1, and Bax, upregulating their expression. It can also suppress expression of anti-apoptotic proteins like BCL-2, BCL-XL, and MCL-1 [379]. As mentioned above, p53 is an unstable and short-lived protein. under normal conditions, an MDM2 E3 ligase, a primary negative regulator of p53, keeps it at a low level due to continuous degradation. MDM2 inhibits p53 activity by ubiquitination, proteasome-dependent degradation, and promoting its nuclear export [380]. The ARF also known as p14^ARF^ in humans and p19ARF in mouse is a tumor suppressor gene derived from INK4a-ARF locus. ARF inhibits MDM2 and prevents p53 degradation. Ectopic MYC expression upregulates ARF. This inhibits MDM2-mediated degradation of p53 and induces expression of p53 directly, which triggers apoptosis [280, 381]. Activated p53 translocates to the mitochondria, interacting with pro-apoptotic proteins and anti-apoptotic members directly [382, 383]. Several studies show that lack of ARF and p53 attenuate MYC related apoptosis, but some groups suggested an alternative pathway for MYC because even in the absence of both ARF and p53, MYC can induce apoptosis [381, 384]. Death-associated protein kinase (DAPK) is a positive mediator of apoptosis activated by MYC and E2F-1. DAP kinase effect on activating p53 is exerted through an ARF-dependent mechanism, which results in p53-induced apoptosis [385, 386]

According to Maclean et al. MYC can increase gamma irradiation (γ-IR)-induced apoptosis by inhibiting BCL-X_L_. In the mouse embryo fibroblasts (MEFs) and Eμ-MYC transgenic mice B cells, MYC functions in synergy with γ-IR to sensitize cells and induce apoptosis independent of p53 [268]. Indeed, MYC does not alone induce the DNA damage response in MEFs but stimulates apoptosis in synergy with γ-IR. MYC along with γ-IR suppress BCL-X gene in the B cells of Eμ-MYC transgenic mice. The loss of BCL-X alone, even without BCL-2, is sufficient to sensitize MEFs to γ-IR induced apoptosis. Finally, activation of MYC can cause a decrease in the steady-state levels of BCL-X_L_ protein by reducing BCL-X transcript and suppressing its promoter activity [268].

Reactive oxygen species (ROS) are reactive chemical species containing superoxide, hydroxyl radical, and hydrogen peroxide with a key role in cell signaling and maintaining homeostasis. Cellular processes, such as metabolism and respiration generate ROS. Excessive ROS can induce apoptosis mediated by mitochondria, death receptors, and the endoplasmic reticulum (ER) [387]. The study conducted by Tanaka et al. determined that in serum deprivation circumstances, overexpression of MYC and E2F-1 inhibit NF-kB activity and suppress superoxide dismutase (SOD). Due to the SOD suppression ROS levels elavete, and cells become vulnerable to apoptosis in serum-deprived conditions [388]. Ornithine decarboxylase (ODC) is another downstream transcriptional target of MYC. ODC encodes the rate-limiting enzyme that catalyzes the first step in the polyamine biosynthesis pathway, converting ornithine to putrescine. MYC stimulates ODC activity to elevate synthesis and catabolism of more polyamine storage. In response to excess polyamine accumulation, polyamine oxidase catabolizes polyamine to ROS and finally induces apoptosis [389].

Initially, in murine B cell lymphoma it was found that FOXO is an antagonist of MYC [390]. FOXO3a is a member of the FOXO protein family that plays a key role in modulating MYC stability and mitochondrial gene expression [391]. Mad/Mxd protein family members are important FOXO3a downstream effectors that dimerize with MYC-associated factor X (MAX) and bind to promoter regions of MYC target genes to block MYC function [392]. FOXO3a can also inhibit MYC activity by enhancing the expression of miRNAs that disrupt translation of MYC mRNA [391, 393]. Taken together, it seems that FOXO3a has an integral role in MYC regulation. In a negative feedback loop, MYC can displace FOXO3a from the promoter of its downstream targets such as GADD45 and PUMA, and downregulates FOXO3a activity [394].

FOXO3a activation leads to a decrease in mitochondrial metabolism and gene expression. As well, FOXO3a reduces ROS production in response to stress. In contrast, MYC elevates mitochondrial output and energy production, promoting cells to re-enter cell cycle. Increased ROS levels cause cell damage [395], and FOXO3a counterbalances this by increasing mitochondrial superoxide dismutase (SOD2) and catalase production [391]. FOXO3a also disrupts the MYC-dependent expression of nuclear encoded mitochondrial genes [396]. Furthermore, activation of FOXO3a, independent of SOD2 activation, alters mitochondrial function and decreases cellular ROS [396, 397]. Overall, the interplay between MYC, FOXO3a, and mitochondrial proteins seems to be critical in regulating MYC and ROS-related activities.

Moreover, cell division cycle 25 (cdc25) phosphatase, a dual-specificity protein phosphatase, is composed of three members: cdc25A, cdc25B, cdc25C. while both cdc25B and cdc25C play an important role in promoting G2\M progression, cdc25A plays a more extensive general function [398]. Cdc25A is a direct transcriptional target of MYC, and its activation contributes to MYC-mediated apoptosis [399]. However, inhibition of cdc25A expression does not suppress MYC-mediated apoptosis because other MYC target genes can compensate for the lack of cdc25A. MYC can stimulate the expression of cdc25A through MYC\MAX heterodimer binding to its promoter. MYC activation can increase cdc25A mRNA and protein levels [147, 400]. The Pim-1 oncogene is another partner for MYC in apoptosis induction. Post-translational phosphorylation of MYC by Pim-1 kinase increases the stability of MYC protein and enhances its transcriptional activity [401]. In addition, Pim-1 can phosphorylate cdc25A as a substrate, and regulates its phosphatase activity. Therefore, evidence indicates that cdc25A links Pim-1 to MYC and plays a vital role in apoptosis induction [402].

## The role of MYC in hematopoiesis and hematological malignancies

MYC is a “global” transcription factor contributing to various cellular processes, one of which is hematopoiesis. Studies have determined that MYC has a quintessential role in nearly every step of the way [23, 24]. The architecture of hematopoiesis is highly organized. Long-term hematopoietic stem cells (LT-HSCs) differentiate into multipotent progenitors (MPPs) first. Both LT-HSCs and MPPs are LSK (Lin^−^/Sca1^+^/c-Kit^+^) cells, which turn into common myeloid and lymphoid progenitors (CMPs and CLPs) [403–405]. Throughout these steps, the self-renewal potential of LT-HSCs is reduced. MYC expression however stays high, indicating its essential role in regulating differentiation and proliferation. High amounts of MYC can be regulated by a single E3 ubiquitin ligase called Fbw7 [406, 407]. The in vitro MYC-mediated inhibition of hematopoietic cell differentiation was first discovered in the 1980s [408]. Later on, a GFP-fused MYC knock-in mouse model was designed to pave the way for MYC-related in vivo studies [409]. The highest MYC expression levels are seen in LSK-originated myeloerythroid progenitor cells, a continuous proliferating cell population [406]. Compared to LT-HSCs, the MYC expression in MPPs is higher [406]. Also, LSK cells of the mice fetal liver shows increased MYC expression during the proliferation period [410, 411], which all are consistent with the study indicating that MYC hinders differentiation in hematopoiesis and consequently propels proliferation [412].

MYC has a role in maturation and expansion of myeloid and lymphoid cells. Initial studies on lymphoid cells showed that the expression of MYC elevates at the transcriptional level during the maturation of pro-B cells into pre-B cells. Thereafter, MYC level would only be increased upon BCR-mediated activation of mature B cells [413–415]. The expression pattern of MYC during T-cell development and the TCR signaling pathway is similar to B-cells [413, 416, 417]. It has been demonstrated that MYC promotes the proliferation of both T and B lymphocytes, as well as synergizing the prolific effects of IL-7 [409, 418].

Compared to the lymphoid differentiation, the myeloid lineage undergoes a more convoluted path. Although MYC involvement in the development of lymphoid cells has received more attention, recent studies have shown that MYC also plays an important role in myeloid cell maturation [419]. The MYC^−/−^ in mice showed not only a diminished lymphocyte production but also demonstrated dysregulated myeloid proliferation, including thrombocytosis, monocyte and neutrophil reduction, and severe anemia [420]. Unlike other cells, megakaryocytes were increased in the MYC^−/−^ model, and despite their small size, they could produce high number of platelets, causing thrombocytosis. This indicates that megakaryocytes are not affected in the same manner as other myeloid cells [420]. All in all, it is not surprising that deregulation of MYC can contribute to tumorigenesis, particularly in hematological cells. In the subsequent sections, we elaborate on the role of MYC in different types of hematological malignancies.

### Role of MYC in lymphocytic leukemia

The first demonstrations of MYC oncogenic capabilities in hematological neoplasms were seen in Eμ-MYC transgenic mice [421–423]. In this model most tumors were developed after 2–5 months, along with mutations in the Arf-Mdm2-p53 pathway [280, 424]. Eμ-MYC transgenic mice models, which overexpress MYC in lymphoid cells mostly develope T-cell lymphoma [425]. Although Burkitt lymphoma is a hallmark of MYC-induced B-cell lymphoma in humans, mice models with induced MYC in their lymphoid lineage could not completely turn to Burkitt lymphoma. To address this, using yeast artificial chromosome (YAC) technology, transgenic mice with 240-kb IgH/MYC translocation region were developed, in which B-cell tumor developed even without Eμ enhancer. This model could mimic B-ALL and Burkitt lymphoma [426–428]. Other mice models were also designed through inserting MYC cDNA into specific regions that transformed mice to Burkitt lymphoma and plasmacytoma model with t(8;14) and t(12;15), respectively [429, 430]. In a more adaptive approach, HSCs derived from fetal liver cells were transduced with either mutant or wild-type MYC-expressing retroviral vectors to produce lymphoma [431]. Although the MYC overexpression has an oncogenic effect, the p53 can counteract it. When the bone marrow (BM) cells with p53^wt/wt^ and null p53^−/−^ phenotypes were transduced with a MYC-encoding retroviral vector, B-cell lymphoma occurred only in p53 null cells [432]. Intriguingly, transduction of BM-obtained p53^−/−^ B-cell lymphoma progenitors by MYC expressing retrovirus, turn the cells into a myeloid lineage in vitro. However, the cells can shift back into B-cell lymphoma upon returning to in vivo environment. This oscillation can be confined via overexpressing Pax5, which maintains the cells in the lymphoid form [433]. Other MYC family members, namely N-MYC, can also contribute to developing AML [434]. Overall, these models have helped to delineate the role of MYC in lymphoid malignancies [435, 436].

Generally, MYC overexpression does not come from mutations in its gene, although some mutations stabilize the MYC protein. Dysregulation of MYC in leukemia and lymphoma, which mostly leads to overexpression, is mainly due to gene amplification, chromosomal translocations, aberrant transcription, and increased stability of mRNA and protein [28]. The high levels of MYC in lymphoid neoplasms mostly confer poor prognosis. It has been revealed that more than 20% of B-ALL patients in different age groups and various demographic backgrounds have MYC overexpression, which implies that routine MYC immunostaining could aid in diagnosing patients at higher risk [437].

#### B-ALL with t(9;22)

The B-cell receptor–ABL proto-oncogene 1 rearrangement reffered to as t(9;22) (BCR-ABL1) occurs in 20–30% of ALL cases. The resulted shorten chromosome 22 is called the Philadelphia chromosome (Ph) [438]. The product of BCR-ABL1 fusion gene is a tyrosin kinase that can induce MYC activation in mice pre-B cells. However, the BCR-ABL1-mediated activation of MYC does not suffice for tumoriogensis. A second hit by oncoproteins such as c-RAF, c-JUN, or RAS is needed for cells to go through a malignant transformation [439, 440]. The suppression of MYC diminishes BCR-ABL1-mediated transformation, meaning that MYC not only possesses a complementary role but also is essential for ensuring the malignant transformation [439, 441]. Moreover, upon pre-BCR activation in lymphoblasts, MYC would be induced via a spleen tyrosine kinase (SYK)-mediated signaling pathway located in its upstream. Subsequently, MYC induction results in increased transcription of BCR-ABL1 in a positive feedback loop to increase the transcription of MYC oncogene [442, 443]. A study reported that MYC oncogene’s transcription could be diminished if SYK is inhibited by small molecules like PRT318 [443]. Parallel to SYK, Sphingosine kinase 2 (SK2) has the ability of acetylating histone H3 within the MYC gene, which induces a MYC-mediated oncogenicity in ALL-mouse models. Inhibiting SK2 or ablating the SK2 gene in murine models can drastically reduce ALL development through reduction in MYC expression and its downstream target genes [444]. In principle, SK2 inhibitors like ABC294640 may be a potential therapeutic approach toward down-regulating MYC expression in different hematological malignancies [445]. Albeit, some clinical trials with SK2 inhibitors have been conducted on diffuse large B-cell lymphoma (DLBCL) (NCT02229981) and multiple myeloma (MM) (NCT02757326).

**Fig. 7:**
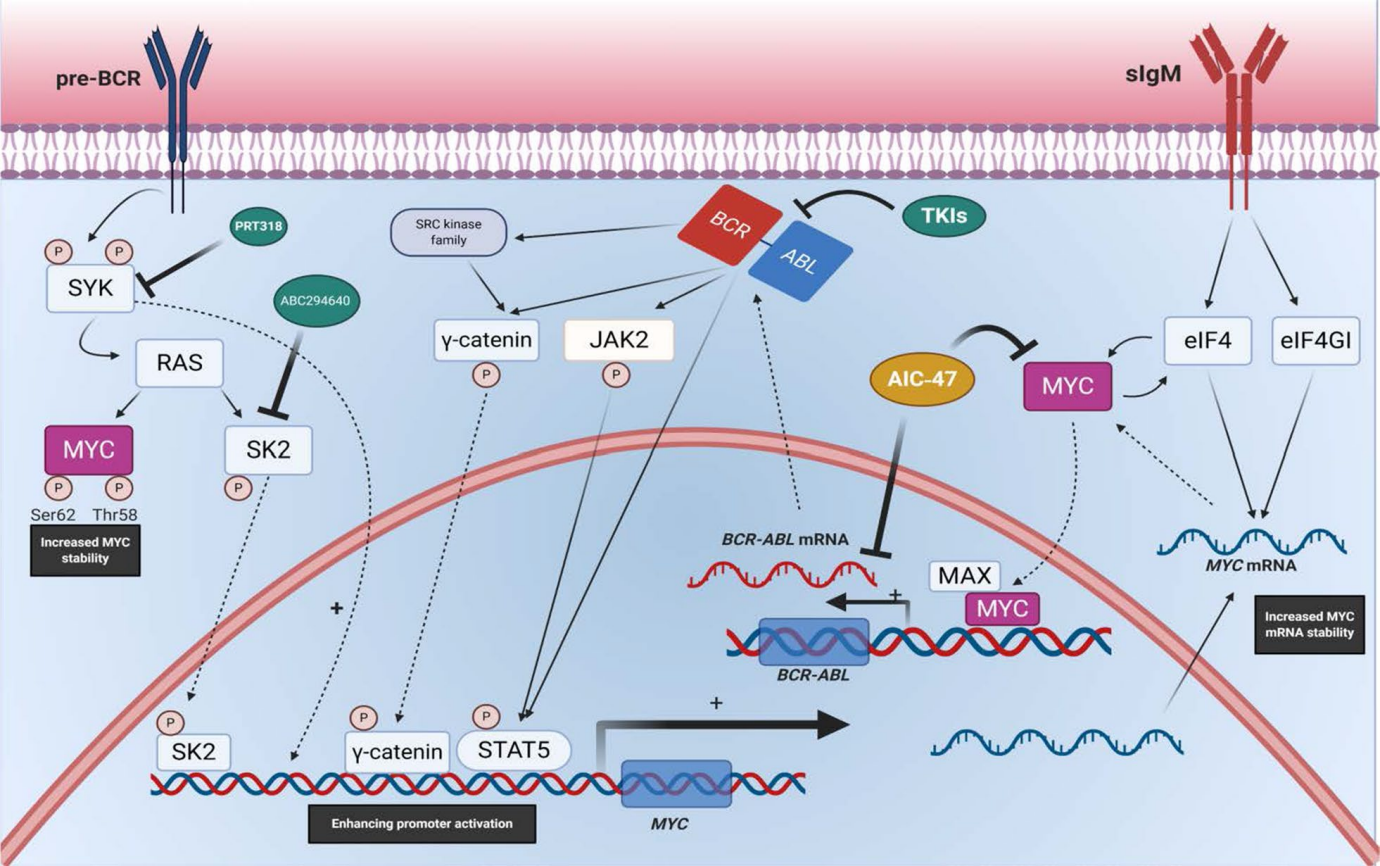
The interactions between BCR-ABL and MYC in B-cell lymphocytic leukemia with t(9;22). Dashed arrows represent an indirect pathway of action, and thick arrows show the mediator's direct effect. Both mature and immature BCR signaling cascades are able to increase the MYC expression. sIgM signaling increases the MYC mRNA stability, which results in a higher level of MYC, and increase the BCR-ABL expression. BCR-ABL can induce MYC expression. Pre-BCR signaling can increase MYC stability and expression. Inhibitors of these signaling mediators can significantly reduce the activity and expression of MYC

A thoroughly studied oncogenic pathway is the Wnt signaling cascade that can promote MYC oncogenicity. The β- and γ-catenins, which are involved in the Wnt signaling pathway [446], can contribute to the pathogenesis of BCR-ABL1-mediated leukemias, including CML and Ph-positive B-ALL [447]. In CML, BCR-ABL1 in HSCs exerts its effects via β-catenin without induction of MYC expression [448]. Contrary to CML, BCR-ABL1 in Ph-positive B-ALL is able to phosphorylate γ-catenin directly and indirectly by SRC family kinase. Subsequently γ-catenin can induce MYC overexpression [447, 448]. On the other hand, following IgM signaling, the MYC mRNA becomes more stable through activation of eIF4 and eIF4GI, supplying higher levels of MYC [449–451]. A positive feedback loop between eIF4 and MYC boosts their activities [452], by which MYC cooperates with MAX, leading to enhanced expression of the BCR-ABL gene [453]. As it was mentioned, BCR-ABL provokes MYC overexpression in a multitude of ways. BCR-ABL1-mediated activation of JAK2 and STAT5 in both CML and Ph-positive B-ALL can sustain elevated MYC levels through promoting its gene expression and guarding MYC against ubiquitination and proteasome-dependent degradation (Fig. 7) [454, 455].

BCR-ABL tyrosine kinase inhibitors (TKIs), are used in treatment of CML and Ph-positive B-ALL patients. Dasatinib, a second generation of TKI, has a dual function against BCR-ABL positive cells and inhibits BCR-ABL and SRC family kinase [456]. However, in some Ph‐positive cases there are mutations that confer resistance to TKIs. A medium-chain fatty-acid derivative named AIC-47 is capable of suppressing BCR-ABL at the transcriptional level and eliminating the Warburg effect [457, 458]. Further, it was demonstrated that AIC‐47 could act as an anti-leukemic agent via down-regulating the MYC regardless of BCR-ABL mutations [459].

#### B-ALL with t(12;21)

The ETV6/RUNX1 (TEL/AML1) rearrangement, as a result of t(12;21) has been reported in 20–30% of childhood B-ALL cases [460, 461]. The MYC oncogenic pathway can synergize with ETV6/RUNX1, leading to promoted MYC oncogenic activity. This is due to a slight homology between the N-terminal region of ETV6 and bHLH region of MYC [28]. The ETV6 can fuse with PAX5 as well and mediates the induction of MYC target genes, leading to ALL progression [462, 463]. In ETV6/RUNX1-rearranged ALL, the MYC gene can be overexpressed by the GTP-binding protein RAC1 [464, 465]. RAC1 enhances the phosphorylation of STAT3, and as a result, MYC expression increases. Therefore, STAT3 inhibitors can enhance in B-ALL cells by decreasing MYC expression [464, 466]. An RNA-binding protein named IGF2BP1 can stabilize the ETV6/RUNX1 rearrangement at the post-transcriptional state, leading to an increased level of MYC [467, 468]. In a recent study, ETV6/RUNX1 fusion in a B-ALL model was knocked out using the CRISPR-Cas9 system, causing an enhanced level of apoptosis and reduced proliferation rate. This can eventually diminish the growth of tumor cells [469]. Abrogating the ETV6/RUNX1 seems to decrease the growth of B-ALL cells and this is due to its role in supporting the oncogenic factors like MYC.

#### B-ALL with the MLL rearrangement

The histone-lysine methyltransferase MLL gene, the so-called KMT2A, is located on chromosome 11 band q23. Chromosomal translocations in this gene frequently occur in both pediatric ALL and AML, representing a considerably poor prognosis in infant leukemia [470–472]. The transloactions involving MYC gene at chromosome 8 are more found in lymphoma comparted to leukemia [471]. It is worth mentioning that the deletion of the CDKN2 gene is frequent in B-ALL patients. It has been shown that t(8;14) is less frequent in cases with wild-type CDKN2 [473]. Furthermore, RS4;11 (KMT2A-AFF1), a B-cell leukemic cell line containing isochromosome i(8q), shows a duplicated MYC gene [474]. The KMT2A-AFF1 rearrangement mostly participates in the leukemic progression as a second hit, and B-ALL patients with this fusion protein possess a significantly promoted MYC gene expression in comparison to patients with AML [475, 476].

Nearly 135 different MLL rearrangements have been found in acute leukemias, which mostly include AF4 (AFF1), AF9, and ELN [477]. The oncogenic activation of the MYC gene mediated by MLL-fusion proteins such as MLL-Fusion/MYC/LIN-28 and MLL-ELN has been reported in B-ALL cases [478, 479]. As mentioned above, p53 can counteract the oncogenic activity of MYC. However, in leukemia cases with MLL rearrangement, MLL protein fusion with USP2 deubiquitinating protein expedites the degradation of p53 by enhancing the promoting USP2 activity. In such cases, MYC activity rises indirectly [480, 481].

The bromodomain and extra-terminal domain (BET) family members are BRD2, BRD3, and BRD4 proteins. These proteins are part of a foundation complex which also includes the super-elongation complex (SEC) and RNA Pol II‑associated factor 1 (PAF1) that all are required for binding of MLL-fused proteins to DNA [482]. The MLL-rearranged proteins bind to a particular complex in the MYC gene's regulatory domains, promoting gene expression at the transcriptional elongation level [483, 484]. Indeed, ChIP and expression analyses have shown that SEC is directly associated with MYC expression in myeloid and lymphoid leukemias [485]. The MLL-SEC rearrangement is stated to be involved in pathogenesis, progression, and metastasis of MLL-rearranged leukemia [486]. Since the BET proteins are therapeutic potential targets, BET inhibitors (iBET) such as iBET-151 and JQ1 (a potent inhibitor of BRD4) were developed to inhibit transcriptional elongation stage of MYC's transcription [483]. 7SK-snRNP complex as the regulator of transcription elongation consists of multiple proteins such as TFEb with kinase activity and HEXIM1, which are positive and negative regulators of transcription elongation, respectively. In order to inhibit TFEb, it is necessary HEXIM1 to interact with other members of 7SK-snRNP complex such as 7SK-RNA. The TEFb is required for initiating the transcriptional elongation of MYC. MYC’s cooperation with BRD4 would be needed if p-TEFb is recruited. This tight interplay between MYC, BRD4, and 7SK-snRNP complex is critical for a fine-tuned transcription elongation [487–490]. The oral iBET Birabresib (OTX015, also known as MK-8628) has been shown to reduce MYC expression and increase HEXIM1 in various types of hematological malignancies [491–493]. The evaluation of OTX015 in AML, ALL, MM, and DLBCL has already passed phase I of a clinical trial (NCT01713582), showcasing promising results [494, 495]. CPI-0610, a BRD4-targeted small molecule can also reduce MYC expression in different types of leukemias [496–498]. It is currently in phase II of a clinical trial (NCT02158858). Additional to agents that indirectly inhibits the MYC activity, there are some direct MYC inhibitors in pre-clinical stage that have been listed in Table 2. For more delineation on clinical trials of MYC inhibitors, a list of clinical trials by these agents in various diseases has also been provided in Table 3.

Overexpression of HDACs such as HDAC9 and SIRT1 have an adverse prognosis in MLL-rearranged lymphoblastic leukemia [499], noteworthy, the latter alters the acetylation of critical genes including TP53, MYC, and NF-kβ, causing drug resistance [28, 499–503]. Indeed, a vast spectrum of HDAC inhibitors (iHDACs) are in development, and some have obtained FDA approval [504]. Contrary to other HDACs, HDAC7 which down-regulates MYC is mostly decreased in various types of leukemia including ones with MLL rearrangement [505]. A study showed that ectopic expression of HDAC7 is potentially anti-oncogenic in B-ALL cells [505]. iHDACs have been used individually and in combination with chemotheraputic agents such as cisplatin, etoposide and azacitidine. Moreover, combination of imatinib with iHDACs has shown promising results in MYC-mediated leukemia [506, 507].

The blood enhancer cluster (BENC), a super-enhancer fragment located within MYC gene governs the oncogenic MYC-expression during the proliferation of B-cell precursors. The BENC confers a vast region of accessible chromatin to transcription factors, enhancing gene expression [508–510]. The adverse MLL–AF9 rearrangement has been reported in both childhood myeloid and lymphoid leukemias [511]. Deletion of BENC demonstrated a drastic reduction of leukemic cells as well as improved prognosis of MLL–AF9-rearranged leukemia [512].

#### Chronic lymphocytic leukemia (CLL)

Chronic lymphocytic leukemia (CLL) is a disease with chronic proliferation of B lymphocytes due to impaired apoptosis and enhanced growth [449, 513]. CLL is a heterogeneous disease with various genetic alterations, such as mutations in immunoglobulin heavy chain variable region (IGHV), MYC translocation, del(11q), del(13q), and del(17p) [514, 515]. MYC translocations are extremely rare in CLL. A comprehensive study evaluated the frequency of MYC translocations in 3405 CLL patients, and showed only a 0.2% (8/3405) occurrence rate [515]. Among them, t(2;8), t(8;22), and t(8;14) were seen in one, two, and five patients, respectively. All CLL patients with MYC rearrangements had poor prognosis, complex cytogenetic abnormalities, and more than 10% prolymphocytes [515]. Some rare CLL cases with MYC translocation have been reported with typical morphology, and proper response to chemotherapy [516, 517]. Another study on 20 patients found that pathogenesis of MYC rearrangements in CLL rely on other genetic abnormalities. For example, complex karyotype is often seen with Richter syndrome transformation, while non-complex karyotype is often associated with proper respons to therapies and achieving remission [518].

Richter syndrome is an aggressive form of CLL and lymphoma associated mostly with molecular aberrations in MYC, CDKN2A/B, NOTCH1, and TP53 [519]. Gain of function mutations of MYC has been shown in 70% of Richter syndrome cases [520]. Despite genetic aberrations in the MYC gene, the MYC hyperactivation in transformed CLL could be as a result of miR-17-92 cluster activity [521], mutations or deletions of MYC regulator MGA [522], and NOTCH1 gene [523], CD40L activation of NF-kβ [524] or BCR (B-cell receptor) signaling [525]. Mutations in CD79 or CARD11 as a part of BCR signaling can provoke chronic BCR signaling in malignant B-cells leading to MYC overexpression [526]. Additionally, the surface immunoglobulin (Ig) of malignant B-cells in some CLL cases is fully glycosylated and mannosylated in the constant and variable regions, respectively. Such an Ig can interact with lectins present in the environment, including DC-SIGN and the mannose receptor [527–529]. Opposite to normal BCR signaling, this Ig can continuously send signals without BCR-antigen endocytosis and upregulates the MYC expression vigorously [527]. Furthermore, the miR-17-92 cluster participates in BCR-mediated upregulation of MYC in which a higher level of MYC leads to induction of miR-17-92, establishing a feed-forward regulatory loop in aggressive forms of CLL [530–532].

The FOXP1 is a transcription factor acting in favor of CLL progression. Although FOXP1 levels should be regulated by miR-150 and miR-34a, the MYC binds to their genes and decreases the gene expression, inducing gain of FOXP1 activity [533, 534]. Fludarabine and doxorubicin are agents capable of swiftly inducing the miR-34a-mediated inhibition of BCR signaling in B-cell neoplasms. These agents are ineffective at hampering BCR signaling in cells with impaired p53 pathway [534, 535].

Unmutated IgHV of BCR, along with heightened surface IgM (sIgM) signaling capability, is associated with high MYC mRNA translation in CLL [450]. Therefore, inhibition of BCR signaling-related factors, BTK and SYK, by Ibrutinib and Tamatinib respectively, can suppress the translational responses [450]. The mutation rate in IgHV depends on the proliferation rate of CLL cells. Cells with accelerated division have less IgHV mutation; in contrast, a higher mutation rate is associated with cells having a slow dividing pattern [536]. Though it is not entirely understood, patients with unmutated IgHV, compared to ones with mutated IgHV, seem to have an inferior survival rate [536, 537]. Mutations in NOTCH1 gene are prominent in CLL cases with unmutated IgHV and intensified sIgM signaling [538]. The gain of function mutations like c.7541_7542delCT in the PEST domain of NOTCH1 gene accumulates and stabilizes the protein, driving an aggressive CLL with high MYC translation [538–540]. Overall, due to cross reaction of NOTCH1 signaling with BCR, use of BCR inhibitor Ibrutinib in CLL could downregulate NOTCH1 activity [541]. This could be a promising approach in downregulation of MYC in CLL.

Various types of BCR signaling inhibitors, such as Ibrutinib, Idelalisib (PI3K inhibitor), Venetoclax (BCL-2 inhibitor), and other novel inhibitors have been developed, and some are approved for standard of care [542, 543]. The BET inhibitors, like the novel GS-5829, target CLL cells, inducing apoptosis via disrupting signaling pathways of MYC, BLK, AKT, and ERK1/2 [544]. Additionally, combinatorial use of BET and BCR inhibitors, have demonstrated further anti-leukemic effects [544, 545]. Despite all the promising results from the standalone targeted- and personalized-therapies, combination chemo-immunotherapy should be used for CLL [537, 546].

#### Lymphoma

Strong evidence showing MYC’s role in human cancer was first found in Burkitt lymphoma [547]. The role of MYC in causing lymphoma was further demonstrated in B-cell and rarely in T-cell lymphoma. [548].

Burkitt lymphoma invariably shows a translocation of MYC gene to the immunoglobulin gene loci [549]. Eighty percent of these translocations are within the immunoglobulin heavy chain gene locus 14q32. Less frequently the immunoglobulin light chain genes, IGκ or IGλ at 2p12 or 22q11 are involved [550, 551]. The translocations result in MYC hypermutation, creating MYC variants with increased oncogenic activity [552–554]. A study suggested that MYC translocation by itself does not cause Burkitt lymphoma, arguing that inhibitor of DNA binding (ID) proteins are another key factor [555]. MYC and ID3 were identified as the most mutated genes in Burkitt lymphoma [556]. Additionally, there is an interplay between BCR, ID3, and MYC in which, upon BCR signaling, MYC and ID3 would be activated. BCR, ID3, and MYC are all in a positive feedback loop [28, 557]. Robust BCR activation requires a functional PI3K. In Burkitt lymphoma, MYC-induced miR-19 inhibits the PI3K inhibitor PTEN, paving the way for lymphomagenesis [531, 555, 558, 559]. PI3K inhibitors may therefore be effective in treating Burkitt lymphoma (Table 3).

MYC rearrangement occurrence rate in DLBCL as the most common type of non-Hodgkin lymphoma is only 10% [549]. Frequent rearrangements in DLBCL involve BCL6 and/or BCL2 (t(14;18)(q32;q21)), which can be seen nearly in 30% of cases [560]. On the other hand, MYC amplification and gain of functions in DLBCL cases come with high MYC copy number and poor prognosis, which indicates an alternative MYC-dependent lymphomagenesis [561]. MYC gene SNPs in DLBCL cases directly correlate with increased cellular proliferation [28].

MYC stability depends on GSK-3β activity. MYC is degraded following GSK-3β-mediated phosphorylation of MYC's Thr-58 residue [562]. In DLBCL, PI3K activation hampers GSK-3β-mediated downregulation of MYC [525]. Furthermore, PTEN, the natural PI3K inhibitor, is often absent in germinal center B-cell-like DLBCL [563]. In a positive feedback loop, MYC upregulation in DLBCL promotes further BCR signaling via recruiting the MIR17HG cluster [532, 564]. These observations imply that inhibitors of the BCR signaling pathway might be effective in blocking this feedback loop.

Based on WHO classification, cases carrying MYC rearrangement accompanied by either BCL2 or BCL6 translocation are designated as double-hit lymphoma (MYC^+^/BCL2^+^ or MYC^+^/BCL6^+^) [565]. Lymphomas bearing all three translocations (MYC^+^/BCL2^+^/BCL6^+^) are known as triple-hit lymphomas [566]. These two types are labelled as high-grade B-cell lymphomas [567]. Additionally, double-hit lymphomas with MYC^+^/BCL2^+^ translocations carrying a TP53 mutation lead to further inhibition of p53-induced apoptosis [568]. Hence, impaired p53-mediated apoptosis, enhanced BCL2-mediated cell survival by BLC2, and promoted MYC-induced proliferation can together form an aggressive phenotype. The first-line treatment for double-hit lymphoma is R-CHOP chemotherapy [565]; however, a recent study demonstrated that targeted therapy using iBET (JQ1, I-BET, and OTX015) alongside BCL-2 inhibitor (ABT-199) could remarkably minimize proliferation and cell survival [569].

Some low-grade lymphomas like follicular lymphoma (FL) can transform to high-grade lymphomas such as DLBCL. FL is a sluggish type of non-Hodgkin lymphoma characterized by the t(14;18)(q32;q21) translocation [570]. In nearly 30% of cases, FL transforms to DLBCL [571]. The transformation of FL to high-grade lymphoma requires an additional hit [572]. Most transformed FL cases have intensified MYC expression in approximately one-fourth of their cells [573]. In addition to MYC, alterations to TP53, CDKN2A, and c-REL are associated with the proliferative phenotype of transformed FL [572, 574]. Like FL, most mantle cell lymphoma (MCL) cases have heightened MYC expression [573]. Contrary to FL, MCL is considered an aggressive malignancy [575]. The genetic hallmark of MCL is the t(11;14)(q13;q32) translocation [576, 577]. MYC rearrangements are usually seen in double-hit MCL [578, 579].

MCL is a heterogeneous disease in which cell-cycle, DNA damage response, and cell survival genes are mostly altered [580, 581]. MCL has two aggressive variants called the blastoid and pleomorphic [580]. In these subtypes, MYC alterations such as t(8;14), t(2;8) and add(8)(q24) accompanied by TP53 alterations lead to a more aggressive MCL [582–584]. Moreover, MALT1 a key factor in MYC stabilization is constitutively expressed in MCL, promoting disease progression.

#### Plasma cell neoplasms

Plasmablastic lymphoma (PBL) is another type of aggressive non-Hodgkin lymphoma, and given the presence of CD138^+^, CD38^+^ and MUM1^+^ it originates from plasmablasts rather than B-cells [585]. PBL is usually found in HIV-positive or immunocompromised cases [586], although there are few studies reporting the occurance of PBL in non-immunodificent patients [587, 588]. Here, BLIMP1-mediated gene repression in the plasmablasts, which is required for plasma cells differentiation downregulates several genes, including PAX5, BCL6 and MYC [589, 590]. It has been reported that 50% of PBL cases bear mutations in BLIMP1 gene (PRDM1), affecting MYC regulation [591]. Intriguingly, 80% of cases have co-expression of BLIMP1 and MYC [591], highlighting a synergistic effect between the two molecules. Almost 50% of PBL cases are found to have MYC rearrangements [592]. Gain of function mutations of MYC are also common in PBL [593]. Loss of p53, has also been shown to contribute to PBL’s aggressiveness [594]. BET inhibitors like JQ1 are capable of inducing cell cycle arrest [482] and may be effective in these cases.

Similar to PBL, plasma cells involved in multiple myeloma (MM) also bear IGH-MYC translocation [595]. Hyperdiploidy, MYC structural variants, and mutations in RAS can also induce MM [595, 596]. A recent comprehensive study showed that the progression of MM heavily depends on MYC, RAS, and NF-κB signaling pathways [595]. Another study on 1342 MM patients showed those with MYC rearrangement had a lower survival rate [597]. There are several MYC targeted therapies for MM that are undergoing clinical trials (Table 3) [598]. Among all the agents, Lenalidomide, which can indirectly target MYC by inhibiting its transcription has gained FDA approval [599].

### Role of MYC in myelocytic malignancies

MYC dysregulates myeloid differentiation [600], and The deletion of MYC in mice diminishes leukomogenesis. The dysregulated myelopoiesis, leads to notable thrombocytosis, drastic monocytopenia and neutropenia, and severe anemia [419, 420]. Several early studies on myeloid leukemia cell lines, such as MEL, K562, and U937, showed that MYC up-regulation could inhibit cell differentiation [601–603]. Nonetheless, in some leukemic cells like NB4 (promyelocytic leukemia cell line), MYC boosts the retinoic acid-induced differentiation [604]. There have been many in vitro- and in vivo- based studies on the role of MYC in myeloid leukemias [419, 605–607]. Nearly all indicated that in both chronic and acute myeloid leukemia, MYC can impact progression and prognosis of the disease.

#### Acute myelocytic leukemia (AML)

AML has various subtypes with a broad spectrum of genetic abnormalities (summarized in Table 1). Unlike lymphoid malignancies, where MYC overexpression is mostly associated with its translocation, the cause of MYC aberrant expression and activity in myeloid malignancies is not thoroughly established [29, 608]. However, a rare recurrent translocation t(3;8) (q26.2;q24), causing MECOM-MYC rearrangement, has been reported to be associated with therapy-related and relapsed AML as well as AML transformed from Ph^+^ CML [609, 610]. Recently, in a detailed and comprehensive study, hotspot mutations in the MYC gene have been identified in AML patients [611]. Other mutations can coincide with MYC mutation in AML. These include: MYC-FLT3, NPM1-MYC, MYC-DNMT3A, NPM1-MYC-FLT3, NPM1-MYC-DNMT3A, and MYC-FLT3-DNMT3A [611]. MYC acts in favor of the progression and maintenance of AML by participating in promoting transcription and translation of the genes involed in cell growth, self-renewal of leukemia stem cell, and chemoresistance [6, 139, 502, 612, 613]. In all subtypes of AML with cytogenetic abnormanlities, MYC overexpression is mainly a sign of inferior overall survival [614]. Since novel MYC-targeting agents show clinical efficiency in AML, understanding the MYC activity in AML is of critical important [615].

**Table 1:** Genetic abnormalities in AML

Type	Biomarker	Fusion protein/role	References
Rearrangements	t(8;21) (q22;q22.1)	AML1-ETO or RUNX1-CBFA2T1	[616]
	inv(16) (p13.1q22) or t(16;16)(p13.1;q22)	CBFB-MYH11	[617]
	t(15;17) (q22;q12)	PML-RARA	[618]
	t(6;9) (p23;q24)	DEK-NUp214	[619]
	inv(3) (q21.3q26.2) or t(3;3) (q21.3;q26.2)	GATA2-MECOM	[620]
	t(1;22) (p13.3;q13.3)	RBM15/MKL1	[621]
	t(9;22) (q43;q11)	BCR-ABL1	[622]
	t(6;11) (q27;q23)	MLL-AF6	[623]
	t(9;11) (p22;q23)	MLL-AF9	[624]
	t(9;11) (p21.3;q23.3)	MLLT3-KMT2A	[625]
	t(6;9) (p23;q34)	DEK-NUp214	[619]
	t(3;8) (q26.2;q24)	MECOM-MYC	[609]
	t(5;11) (q35;p15.5)	NUP98-NSD1	[626]
Mutations	FLT3, KRAS, NRAS, KIT, PTPN11, NF1	Signaling mediators	[627]
	DNMT3A, IDH1/2, TET2, ASXL1, EZH2, MLL/KMT2A	Epigenetic mediators	[627]
	CEBPA, RUNX1, GATA2	Transcription factors	[628]
	TP53	Tumor suppressing factor	[629]
	SRSF2, U2AF1, SF3B1, ZRSR2, RBM25	Spliceosome complex	[630]
	NPM1	Nucleophosmin	[631]
	RAD21, STAG1, STAG2, SMC1A, SMC3	Cohesin complex	[632]
	MYC	Proto-oncogene	[633]

Inducing leukemogenesis through overexpressing MYC, is a common approach to study the effects of this proto-oncogene. MYC expression alone does not suffice for the transition of cells to AML; in fact, continuous co-stimulation with IL-3 and GM-CSF are also required, suggesting the vital role of microenvironment and cytokines in the development of MYC-mediated AML [634]. Most recently, leukemic microenvironment-originated exosomes have attracted significant attention. This is due to their ability to carry cargos like MYC between tumor cells, inducing leukemic progression [635]. Investigating leukemia-related exosomes seems beneficial to a better understanding of leukemogenesis, leukemia diagnosis, and efficient therapeutic strategy.

The fine-tunned balance between pro- and anti-apoptotic signals are mostly dysregulated during tumorigenesis. All six members of the anti-apoptotic BCL family, including BCL2, BCLxl, BCLw, BCLb, BFL1, and myeloid cell leukemia 1 (MCL1), have been reported to advance the MYC-induced myeloid leukemogenesis [636]. Results from MYC-induced AML in mice have shown presence of highly expressed anti-apoptotic protein MCL-1 [637]. MCL-1 inhibitors such as AZD5991 (NCT0321868), S64315 (NCT0297936), AMG176 (NCT0267545), and AMG397 (NCT03465540) are under evaluation in phase 1 clinical trials for AML patients.

Aberrant transcription factors encoded by AML-related rearrangements such as AML1-ETO (RUNX1-CBFA2T1), PML-RARα, and ZBTB16 (PLZF)-RARα have been reported to induce Wnt signaling, leading eventually to the upregulation of MYC [638–640]. The Wnt-induced upregulation of MYC in AML with RUNX1-CBFA2T1 fusion protein is associated with a feed-forward loop between RUNX1-CBFA2T1 and γ-catenin. The RUNX1-CBFA2T1 elevates γ-catenin expression and mediates the Wnt-induced MYC upregulation [638]. RUNX1-CBFA2T1 can also induce MYC upregulation by provoking the expression of β-catenin, another Wnt family member [638]. In addition to RUNX1-CBFA2T1, LEO1, a direct and specific substrate for phosphatase of regenerating liver-3 (PRL-3), can bind to β-catenin and increase its activity. This results in transactivation of the MYC gene [641]. Nearly 43% of AML cases are PRL-3^+^ in which they have exhibited sensitivity to β-catenin inhibition. This shows AML-PRL-3^+^ is dependent on Wnt signaling [641, 642]. A study found that Kangai 1 (KAI1), also called CD82, is overexpressed in pediatric AML cases. This activates the Wnt/β-catenin pathway and its target MYC, supporting the proliferation of leukemic cells and -chemoresistance to doxorubicin. Remarkably, the knocking-down of CD82 led to apoptosis and repressed growth and reduced chemotherapy resistance in AML cells [643]. Apart from Wnt signaling, proliferation and self-renewal in AML cells with RUNX1-CBFA2T1 significantly depends on TATA-Box binding protein associated Factor 1 (TAF1) [644]. Due to the considerable overlapping of binding sites of TAF1 and RUNX1-CBFA2T1, the knocking down of TAF1 or inhibiting it by Bay-364 can impair the MYC expression and promote differentiation and apoptosis in leukemic cells [644].

Protein phosphatase 2A (PP2A) endogenous inhibitor, ARPP19, might be a potential biomarker for patients with AML as it associated with the induction of MYC overexpression [645]. Based on the ELN risk group classification for AML, in both favorable- and intermediate-risk groups, a high level of ARPP19 increases the necessity of BM transplantation while patients with low levels of ARPP19 can mostly be cured with chemotherapy [645].

Furthermore, FLT3 mutations are found in AML patients. These include internal tandem duplication (ITD) and tyrosine kinase domain (TKD) mutations [646]. These mutations can mediate ligand-independent activation of the canonical Wnt/β-catenin signaling pathway, resulting in the upregulation of MYC and myeloid transformation [647]. Using merely FLT3 tyrosine kinase inhibitors might not be efficient to halt AML progression. This is due to MYC-mediated stabilization of the histone deacetylase SIRT1 which causes treatment resistance in AML [502]. However, strategies such as disrupting Wnt/β-catenin signals or combining Pim-1 kinase inhibitors or PP2A activators with FLT3 inhibitors synergize and promote their anti-leukemic effects in AML [648–650]. Apart from signaling pathways, dysregulation of mRNA splicing is also found in AML, where the splicing regulator RBM25 is reduced [651]. Decrease in RBM25 increases MYC levels followed by enhanced proliferation along with reduced apoptosis in leukemic cells [651].

Cancer cells often utilize double minutes (dmin), homogeneously staining regions (hrs), and ring chromosomes to do extra-chromosomal gene amplification [652]. This mechasnism of gene amplificationoccasionally happens in leukemias [653]. In this manner, two of the most amplified AML-related genes are MYC and MLL [654, 655],which the presence extra-chromosomal gene amplifier implies a poor prognosis although mechanism of action has not been elucidated [656].

#### Myeloproliferative neoplasms (MPNs)

MPNs are associated with the proliferation of one or more members of the myeloid lineage. There are mainly two categories based on presence of Ph chromose: Ph (BCR-ABL)-positive CML and Ph-negative group including essential thrombocythemia (ET), polycythemia vera (PCV), and primary myelofibrosis (PMF). In the Ph-negative group the involvement of JAK2, MPL, and/or CALR aberrations are frequent [657, 658]. However, the co-occurrence of Ph-positive and other aberrations have been infrequently reported in CML [657].

The aforementioned feed-forward interplay between MYC and BCR-ABL thoroughly describes the BCR-ABL-mediated upregulation of MYC in both CML and Ph-positive ALL. Furthermore, BCR-ABL can mediate the phosphorylation of Ser62 residue of MYC, though dasatinib can induce its dephosphorylation [659]. Progression of CML into blastic crisis is associated with a higher MYC expression level. This correlates with inferior response to imatinib and poorer prognosis [605]. In CML patients who further progress to the blastic crisis phase, endogenous PPA2 inhibitor, CIP2A increases prior blastic phase. CIP2A blocks dephosphorylation of MYC at Ser62 residue [660, 661]. In the blastic phase, the MYC target genes, including the ATP-binding cassette (ABC) transporters, are up-regulated. However, they might play a role in resistance to imatinib [662]. It has been suggested that TKIs can be combined with other therapeutic strategies to overcome ABC-related drug resistance [663].

Fbxw7-mediated ubiquitination of MYC is considered an essential regulatory step in CML [664]. The Fbxw7 mediate decrease in proliferation, survival and maintenance of leukemia-initiating and leukemia stem cells (LIC/LSC). Upon phosphorylation of MB-Box I domain of MYC at Thr-58 and Ser-62, Fbxw7 targets MB-Box I domain and destabilizes the MYC [665]. Phosphorylation of Thr-58 relies on the earlier phosphorylation of Ser-62. Intriguingly, The sole phosphorylation of Ser-62 stabilizes the MYC, whereas further phosphorylation of Thr-58 propels MYC toward ubiquitination and degradation [59]. The BCR-ABL fusion protein is not solely required for LSC survival; thereby, TKIs fail to annihilate LSCs responsible for CML maintenance [666]. To address this impediment, a study recommended an intriguing approach without application of BCR-ABL inhibitors. In this dual approach p53 was activated and MYC was inhibited. This resulted in synergetic extermination of cells, further differentiation, and almost obliteration of transplantable human LSCs in mice,whilie healthy HSCs were spared [667]. This might be a potential strategy in TKI resistance and relapsed patients.

The prevalence of JAK2 V617F mutation in MPNs, including PCV, ET, and PMF, is approximately 90%, 50%, and 50%, respectively [668]. Generally, the signaling cascade initiated by JAK2/STAT5 can mediate MYC expression. In MPNs with JAK2 V617F mutation however, the signaling is independent of any ligand [669]. Hyperactivation of mutated JAK2 needs an intact FERM domain to induce MYC overexpression [670]. Both PIM and JAK2 inhibitors have been used to downregulate MYC and repress MPN cell proliferation; however, combining them can overcome MPN drug resistance and synergistically enhance their suppressing effect upon MYC [671]. The above mentioned strategy implemented by Abraham et al. in which p53 is provoked and MYC is inhibited, has also been suggested as an effective therapeutic approach for JAK2-mediated MPNs [667, 669].

## MYC inhibitors

MYC is a well-established oncogene that can be targeted by inhibitors. The table below provides a list of MYC inhibitors in the pre-clinical stage.

### Direct MYC inhibition

Studies have pointed that direct MYC inhibition brings about prompt tumor regression, highlighting the importance of this approach [691]. Inhibiting MYC/MAX dimerization and E-box binding by peptides and small molecules, as well as using RNA interferences (miRNA, siRNA) downregulating MYC translation, can directly block MYC activity [691].

Recently, OmoMYC agents as MYC dominant-negative proteins have attracted great attention [683]. Since MYC is a master transcription factor, blocking it by OmoMYC at first seemed to be challenging due to the expected side effects. However, after testing on animal models, side effects have shown to be mild, well-tolerated, and reversible [692]. Intriguingly, OmoMYC could infiltrate cells, inhibiting MYC activity by its spontaneous cell-penetrating ability. Moreover, using OmoMYC through direct tissue delivery and systemic administration in non–small cell lung cancer models showed significant therapeutic potential [692]. OmoMYC can inhibit MYC by two mechanisms; interrupting MYC/MAX dimerization and E-box binding [693]. There are different types of OmoMYC that are in pre-clinical phases. Among them, OMO-103 [NCT04808362] and OMO-1 [NCT03138083] have made it to the clinical trials (Table 3).

In addition to OmoMYC, other compounds can also inhibit MYC/MAX dimerization (Table 2). The MYC/MAX destabilizers, IIA6B17, 10058F4, and 10,074-G5, were extracted from a peptidomimetic library [672, 694]. It seems that IIA6B17 can act against c-Jun, due to the resemblance of its leucine zipper structure [695]. JY-3-094 was found to be able to hinder proliferation in MYC overexpressed cells (HL60 and Daudi cells) via inhibiting MYC/MAX dimerization [696]. If a phenyl ring is added to JY-3-094 the result would be a MYC inhibitor called 3jc48-3 with five times more potential in arresting the cell cycle at G1/G0 [696]. Such significant potential in inhibiting MYC/MAX is thought to be the effect of phenyl ring on F375, I381 and R378 residues in the MYC/MAX dimer [696, 697].

**Table 2:** List of pre-clinical direct MYC inhibitors

Mechanism	Type	Compound	References
Inhibitor of MYC/Max dimerization	Small Molecule	IIA6B17	[672]
		10058-F4	[673]
		10074-G5	[674]
		JY-3-094	[675]
		3jc48–3	[676]
		MYCro1, MYCro2, MYCro3	[677]
		MYCMI-6	[676]
		KJ-Pyr-9	[678]
		EN4	[679]
		MYCi361	[680]
		MYCi975	[681]
		KI-MS2-008	[682]
	(Poly)peptide	OmoMYC	[683]
		FPPa-OmoMYC	[684]
		Max bHLHZ (OmoMYC)	[685]
		Mxd1	[686]
		Monoclonal antibody	[687]
		H1 peptide	[688]
Inhibitor of E-box binding	Small Molecule	JKY-2-169	[689]
	(Poly)peptide	OmoMYC	[683]
		Max bHLHZ	[685]
		Mxd1	[686]
		ME47	[690]

KJ-PYR-9, a compound derived from Kröhnke pyridine library were found to have anti-DNA-binding and anti-MYC/MAX dimerization features [698]. The KJ-PYR-9 effects on inhibiting proliferation in xenografts bearing MYC-amplified human cancer cells seem promising [698]. Other small-molecules with properties like KJ-PYR-9 are MYCro1, MYCro2, and MYCro3 [677, 699]. MYCro3, in combination with Palbociclib, a CDK4/6 inhibitor, has shown great potential in treating HER-2 negative metastatic breast cancer [700], indicating that the direct MYC inhibition can synergically increase the effect of other targeted therapies.

Most recently, based on bimolecular fluorescence complementation, another direct MYC inhibitor, called MYCMI-6 was identified among 2000 agents, which could interrupt the MYC/MAX dimerization [701, 702]. Recent studies have demonstrated that the effect of MYCMI-6 on breast cancer in inducing cell-growth inhibition and apoptosis [678, 703]. EN4 is also a novel covalent small molecule, directly targeting C171 residue of MYC, causing thermal destabilization of MYC and MAX, as well as disrupting MYC transcriptional activity. Overall, the EN4 features enable it to block tumorigenesis [680]. Han et al. discovered two compounds (MYCi361 and MYCi975) capable of phosphorylating Thr-58 residue of MYC, propelling it toward proteasome-mediated MYC degradation [704]. MYCi975 is an enhanced model of MYCi361. Results of in vivo MYCi-induced tumor regression capacity are shown to be promising since it enhances infiltration of immune cells into tumor microenvironment, upregulates PD-L1 on tumor cells, and synergies with anti-PD1 immunotherapy [681, 704].

Stabilization of MAX homodimer by an asymmetric polycyclic lactam termed KI-MS2-008 is another approach toward attenuating MYC/MAX dimerization, resulting in the reduction of MYC protein and the expression of its target genes. Also, in vivo evaluations show that KI-MS2-008 abrogates the ability of tumor cells to grow properly [682]. MAX homodimer stabilizers like KI-MS2-008 could also be utilized alongside monoclonal antibodies against PD-1 or PD-L1 immune checkpoints to synegies the antitumoral effects [705]. Moreover, there is a protein called MXD1 that can couple with MAX, afterwards hijacking E-box of MYC target genes, and antagonizing MYC transcriptional activity [706]. Mad can act similar to MXD1, and it is claimed that Mad is tenfold more potent compared to OmoMYC [686]. A small hybrid protein named ME47 can also inhibit MYC transcriptional activity by seizing the E-box binding site of MYC target genes, resulting in a significant reduction in cell proliferation of tumor xenografts [690]. On the contrary, JKY-2-169 binds to MYC-MAX heterodimer, not allowing it to bind with DNA E-box, without affecting MYC-MAX formation. This JKY-2-169-mediated perturbation of DNA binding has been shown to reduce MYC-induced cell proliferation, cell cycle arrest, and apoptosis [707, 708].

Monoclonal antibodies against MYC can also inhibit its activity. Park et al. showed that these antibodies are capable of targeting MYC and MYC-MAX heterodimer [687]. For antibodies intracellular infiltration remains a challenge. There is a small alpha-helix MYC inhibitor peptide called H1 that can be carried to the nucleus via particular non-toxic carriers and decrease the MYC-MAX dimerization, reducing the expression of MYC target genes [688].

Using siRNAs for in vivo inhibition of MYC translation is another approach, although transporting siRNAs into cells requires reliable carriers. DCR-MYC, an EnCore lipid nanoparticle containing siRNA against MYC, was used in clinical trials to treat solid tumors [NCT02314052] and hematological malignancies[NCT02110563] [691, 709]. Further studies however showed side effects like thrombotic microangiopathy, which terminated the clinical trials [691, 710]. Studies on MYC-targeted siRNAs are still going on.

There are miRNAs such as miR-494, which targets MYC translation. It has been shown that miR-494 is downregulated in ovarian cancer, whereas overexpression of miR-494 hampered the growth of the cancer cells and limited their migration [711]. Additionally, ectopic miR-494 overexpression inhibits the proliferation of pancreatic cancer cells via inducing apoptosis, cell-cycle arrest, and senescence, which remarkably prohibited the invasiveness of the cancer cells [712]. This particular miR has recently undergone clinical trials. A MYC-targeted phosphorodiamidate morpholino oligomer (PMO) called AVI-4126 can prohibit ribosomal assembly, therefore inhibiting MYC translation [677, 713]. AVI-4126 has been extensively applied to various cancers, and results were shown promising, which led this particular PMO to clinical trials.

### Indirect MYC inhibition

MYC inhibition indirectly by targeting its regulating factors provides a more flexible approach toward MYC inhibition. MYC regulating factorsinclude BET family, MCL-1, BCR-signaling mediators, HDACs, PI3Kδ, DNA-PK, CDKs, kinases and G-quadraplex (Refer to “The role of MYC in hematopoiesis and hematological malignancies" section). There are various inhibitors for the aforementioned MYC regulators; among them, some have made it to clinical trials (Table 3).

**Table 3:** Clinical trials targeting MYC

Type	Mechanism	Condition(s)	Compound	Phase	NCT number
Direct MYC inhibition	siRNA against the MYC	Hepatocellular Carcinoma	DCR-MYC	Phase 1 Phase 2	NCT02314052
	Inhibits MYC/MAX dimerization Inhibition of E-box binding	Advanced Solid Tumors Non-small-cell lung carcinoma (NSCLC) Triple-negative Breast Cancer	OMO-103	Phase 1 Phase 2	NCT04808362
	Inhibits MYC/MAX dimerization	Neoplasms	OMO-1	Phase 1 Phase 2	NCT03138083
	Downregulation of MYC	Ischemic Stroke	miR-494	-	NCT03577093
	Interrupts the translation of MYC gene	Neoplasms	AVI-4126 (RESTEN-NG)	Phase 1	NCT00343148
Indirect MYC inhibition	Alteration of MYC translation (BET Bromodomain inhibitors)	Castration-Resistant Prostate Carcinoma Metastatic Prostate Adenocarcinoma Metastatic Prostate Small Cell Carcinoma Stage IV Prostate Cancer AJCC v8 Stage IVA Prostate Cancer AJCC v8 Stage IVB Prostate Cancer AJCC v8	ZEN-3694	Phase 2	NCT04471974
		Solid Tumor Lymphoma Brain Tumor	BMS-986158	Phase 1	NCT03936465
		Lymphoma, Non- Hodgkin	CC-95775 (FT-1101)	Phase 1	NCT04089527
		Diffuse Large B-cell Lymphoma (DLBCL) High-Grade B-cell Lymphoma	RO6870810	Phase 1	NCT03255096
		Neoplasms	GSK525762	Phase 2	NCT01943851
		Myelofibrosis Primary Myelofibrosis Post-polycythemia Vera Myelofibrosis Post-essential Thrombocythemia Myelofibrosis	CPI-0610	Phase 3	NCT04603495
		Metastatic Malignant Solid Neoplasm Recurrent Malignant Solid Neoplasm Recurrent Platinum-Resistant Ovarian Carcinoma Refractory Ovarian Carcinoma	ZEN-3694	Phase 1	NCT04840589
		Malignant Solid Tumors Lymphoma Ovarian Cancer Breast Cancer Pancreatic Cancer Prostate Cancer	AZD5153	Phase 1	NCT03205176
		Neoplasms NUT Carcinoma	BI 894999	Phase 1	NCT02516553
		AML Including AML de Novo and AML Secondary to MDS DLBCL	Birabresib (MK-8628, OTX015)	Phase 1	NCT02698189
	MCL-1 inhibitor	Relapsed or Refractory AML	AZD5991	Phase 1 Phase 2	NCT03218683
		AML	S64315 (MIK665)	Phase 1 Phase 2	NCT04629443
		Relapsed or Refractory Multiple Myeloma Relapsed or Refractory AML	AMG 176	Phase 1	NCT02675452
		Multiple Myeloma Non-Hodgkins Lymphoma Myelodysplastic Syndrome	AMG 397	Phase 1	NCT03465540
	Inhibiting BCR-signalling	Prolymphocytic Leukemia Recurrent Adult Diffuse Large Cell Lymphoma Recurrent Mantle Cell Lymphoma Recurrent Small Lymphocytic Lymphoma Refractory Chronic Lymphocytic Leukemia	Ibrutinib	Phase 1	NCT02303392
		Lymphoma, B-Cell Small Lymphocytic Lymphoma CLL Waldenstrom Macroglobulinemia Mantle Cell Lymphoma Diffuse Large B Cell Lymphoma Richter's Transformation Follicular Lymphoma Marginal Zone Lymphoma	ARQ 531	Phase 1 Phase 2	NCT03162536
	Epigenetic silencing (HDAC inhibitors)	Diffuse Large B-cell Lymphoma	Tucidinostat	Phase 3	NCT04231448
		Relapsed and refractory lymphoma	Entinostat	Phase 2	NCT03179930
	PI3K inhibitor	B Cells-Tumors B Cell Chronic Lymphocytic Leukemia Follicular Lymphoma Mantle Cell Lymphoma Large B-Cell Diffuse Lymphoma of Bone (Diagnosis)	Idelalisib	Phase 1	NCT03151057
	Dual inhibitor of PI3Kδ and CK1ε	CLL B-cell Non-Hodgkin Lymphoma	TGR-1202	Phase 1	NCT03283137
	Dual inhibitor of PI3Kδ and DNA-PK	Diffuse Large B Cell Lymphoma Follicular Lymphoma CLL Small Lymphocytic Leukemia B Cell Lymphoma Marginal Zone Lymphoma Waldenstrom Macroglobulinemia Peripheral T Cell Lymphoma	BR101801	Phase 1	NCT04018248
	Dual inhibitor of PI3Kδ and HDACs	Relapsed and/or Refractory DLBCL With MYC Alterations	Fimepinostat (CUDC-907)	Phase 2	NCT02674750
	Inhibitor of CDK1, CDK2, CDK5 and CDK9	Advanced or Metastatic Breast Cancer Triple Negative Breast Cancer	Dinaciclib	Phase 1	NCT01676753
	CDK9 inhibitor	Relapsed Solid Tumors Refractory Solid Tumors Non-Hodgkin Lymphoma	KB-0742	Phase 1	NCT04718675
	Multi kinase inhibitor: inhibits CDKs 1, 2, 7 and 9 together with JAK2 and FLT3	AML ALL Blast Crisis MDS Multiple Myeloma	TG02	Phase 1	NCT01204164
	G-quadraplex stabilizer at MYC promoter	AML High Risk Myelodysplasia	APTO-253	Phase 1	NCT02267863
		Advanced Solid Tumors Lymphoma	CX-3543	Phase 1	NCT00955786

#### BET family inhibitors

BET family includes BRD2, BRD3, and BRD4 proteins. BET proteins recognize the acetyl-lysine residues of histones, recruiting transcription factors, especially the MYC oncoprotein, to promote gene expression. BET inhibitors (iBETs) were found to be effective in blocking oncoproteins and decreasing tumorigenesis. The first iBET entering clinical trials was Birabresib (MK- 8628, OTX015) [492]. Due to the safety, efficacy, and pharmacokinetics of Birabresib in hematological and solid tumors, phase II clinical trial has been recommended [714].

Among different iBETs, ZEN-3694 is an orally administered pan-BET inhibitor. Currently, ZEN-3694 is under clinical trial in phase I and II. Drug-resistance to some targeted therapies seem to be inevitable, due to the overexpression of master oncogenes like MYC. Using ZEN-3694, alongside Enzalutamide as an inhibitor of androgen receptor for treatment of prostate cancer, synergically enhances the effect of Enzalutamide [715, 716]. FT-1101 (CC-95775) can also act as pan-iBET. A study on the effect of FT-1101 on various human leukemia cell lines exhibited higher inhibition of proliferation in tumor cells compared to JQ1. Monotherapy with FT-1101 displayed tolerance and proper safety [717, 718].

BMS-986158 is another orally bioavailable BET inhibitor, shown to be well-tolerated in treatment of advanced cancers. The sole reported side effect was reversible thrombocytopenia [719, 720]. Among iBETs, BMS-986158 has notable pharmacodynamics profile with a longer half-life.[721]. RO6870810 (also termed RG6146 and TEN-0) is a novel iBET, resembling the JQ1 class. However, it outperforms JQ1 in solubility, metabolic stability, and binding to serotonin receptors. Additionally, alpha assay technology shows the remarkable affinity of RO6870810 toward the acetyl-lysine recognition pocket of the BET family [722, 723].

In (nuclear protein of the testis) NUT carcinoma, the BET family would join the NUT if NUTM1 rearrangements occur. The fused oncoproteins alter MYC regulation. A novel iBET RO6870810 (also known as RG6146 and TEN-0) can dissociate the BRD-NUT oncoproteins from DNA, inhibiting the proliferation of cancer cells. RO6870810 has also been tested on DLBCL where MYC is associated with the aggressiveness of DLBCL [722]. The clinical potential of RO6870810 is now being evaluated in clinical trials.

Recently, Molibresib (GSK525762), an orally administered iBET, has also been tested on NUT carcinoma. Phase I clinical trials assessed safety, tolerance, pharmacokinetics, and pharmacodynamics. The preliminary results recommended moving to phase II [724]. In addition to NUT carcinoma, GSK525762 has also been tested on a vast spectrum of hematological neoplasms in order to indirectly inhibit the master oncoprotein MYC. Minor dose-limiting toxicity related to GSK525762 has been seen in AML patients, including diarrhea and reduced ejection fraction, although both were reversible. Overall, despite seeing a complete response to GSK525762, due to some adverse effects, the phase one clinical trials recommended further investigations [615].

Inhibition of BET family members in hematological neoplasms has shown to be a quite effective treatment approach. Among other iBETs, CPI-0610 has been evaluated in clinical trials for primary myelofibrosis, post-polycythemia vera myelofibrosis and lymphomas [498, 725, 726]. Using CPI-0610 solely or combined with a Janus kinase 1/2 inhibitor (Ruxolitinib) on refractory or intolerant advanced myelofibrosis demonstrated promising efficacy in patients with inadequate responses to Ruxolitinib. This seems to be the effect of MYC inhibition, enhancing Ruxolitinib effects. Following treatment, the patients exhibited improvement of bone marrow function [725]. CPI-0610-mediated MYC inhibition induces G1 arrest and apoptosis in multiple myeloma resulting in tumor regression. Moreover, CPI-0610 alongside immunomodulatory drugs for multiple myeloma such as thalidomide, lenalidomide and pomalidomide can be synergically utilized in multiple myeloma treatment [497].

BRD4 could be targeted by AZD5153, an orally bioavailable iBET. Unlike other monovalent iBETs, the AZD5153 is a bivalent inhibitor that results in further antitumor activity. The effect of AZD5153 on AML, multiple myeloma, and DLBCL xenografts has been reported to be significant. The AZD5153 modulates MYC, E2F, HEXIM1 and mTOR pathway, indicating the remarkable potential of this iBET. Of note, AZD5153-mediated alteration of mTOR positively enhances the effects of AZD5153 on tumor cells [727, 728]. Overexpressed MYC and BCL2 in double-hit lymphoma and double expressing lymphoma has poor prognosis. Utilizing AZD5153 could downregulate several oncogenic factors, including MYC and B-cell development-related factors. Notworthy, AZD5153 neither could downregulate BCL2 family members (anti-apoptotic factors), or induce activation of BH3-only proteins (pro-apoptotic factors). AZD5153 and BCL2 inhibitor (AZD4320) synergistically induced antitumor effects [729]. This potent iBET is now under clinical trials for the treatment of various diseases, including malignant solid tumors [NCT03205176]. Similar to AZD5153, another iBET called BI894999 can also inhibit BRD4 leading to modulation of MYC and HEXIM1 in AML cells. This particular iBET, in combination with CDK9 inhibitor, shows an expedited apoptotic response due to the reduction in global p-Ser2 RNA polymerase II levels [730].

#### MCL-1 inhibitors

All members of the anti-apoptotic BCL family, especially MCL1, have been reported to advance the MYC-induced myeloid leukemogenesis [636]. Results from MYC-induced AML in a mouse model have shown presence of highly expressed anti-apoptotic protein MCL-1 [637]. High levels of MCL-1 provoke tumorigenesis and drug resistance, indicating the potential of MCL-1 inhibitors as a therapeutic option.

Accordingly, AZD5991, a selective small-molecule targeting MCL-1 could be a potential choice in AML treatment. Due to the significant potential in inducing prompted Bak-dependent apoptosis and high antitumor activity in pre-clinical studies on myeloma and AML, AZD5991 has been chosen to enter clinical trials as a treatment for relapsed or refractory AML. It has been used both as a monotherapy and combined with Bortezomib (inhibitor of the 26S proteasome) or Venetoclax (BCL-2 inhibitor) [731]. S64315 (MIK665), a highly selective MCL-1 inhibitor can act partly similar to AZD5991 by inhibiting MYC activity and inducing Bax/Bak-mediated apoptosis. S64315 is a well-tolerated compound capable of inducing dose-dependent apoptosis, demonstrating potent antitumor activity in hematological malignancies such as AML, multiple myeloma, and lymphoma [732]. Since BCL-2 family members and MYC act cooperatively in cancers [733], inhibiting MCL-1 could be a potential approach for MYC-involved cancers.

AMG class of MCL-1 inhibitors has recently entered phase I clinical trials as a treatment for various hematological malignancies, including relapsed or refractory multiple myeloma. [NCT02675452, NCT03465540]. AMG176 was introduced to be administered intravenously, and AMG397 was developed as the first orally bioavailable MCL1 inhibitor. AMG397 compared to AMG176 has improved potency and pharmacokinetic features [734]. It selectively competes with other BCL family members over the BH3-binding site of MCL1 and elevates the caspase 3/7 activity. It is reported that hematological neoplasms are highly sensitive to this particular compound [734].

#### BCR-signaling inhibitors

BCR signaling can activate several transcription factors, especially MYC. BCR-signaling mediators, such as BTK are involved in inducing MYC during B-cell development [442, 443]. Ibrutinib, a BCR-signaling inhibitor which is under clinical trials for several hematologic malignancies could be a potential compound for inhibition of MYC induced proliferation in B-cell malignancies [NCT02303392]. It is worth mentioning that in malignancies like MCL, where one-third of cases do not respond to Ibrutinib, MYC is overexpressed, suggesting that MYC can block Ibrutinib activity [735]. The underlying mechanism responsible is MYC-induced BTK overexpression [736].

ARQ531 is a novel BTK inhibitor that can also act against other BCR signaling factors like SRC kinases and ERK-signaling pathway. Targeting a multitude of BCR-signaling related factors in CLL models with ARQ531 showed robust inhibitory potential in treatment of Ibrutinib resistance cells [737]. Due to the great potential of ARQ531 in overcoming resistance to Ibrutinib, it has entered phase I clinical trials for various MYC-involved hematological neoplasms [NCT03162536].

#### HDAC inhibitors (epigenetic modulators)

Histone deacetylase family members play an essential role in regulating the MYC level [738]. For instance, SIRT1 is a class III histone deacetylase responsible for the acetylation of critical genes, including TP53, MYC, and NF-kβ [28, 499–503]. On the other hand, HDAC7 that is mostly decreased in various types of leukemia can downregulate MYC[505]. Taken together, it seems that HADCs are vastly involved in MYC regulation; thus, inhibition of HDACs provoking MYC activity can be greatly beneficial for the treatment of MYC-involved cancers.

HDAC inhibitor Entinostat (class I iHDAC) is capable of inhibiting HDAC2, a partner of MYC in medulloblastoma. Entinostat-mediated inhibition of HDAC2 reduces MYC transcriptional activity, and hinders MYC-DNA binding, indicating the efficiency of iHDACs [739]. Entinostat has thoroughly been studied on hematological malignancies [740], and now is in phase II clinical trial for relapsed and refractory lymphoma [NCT03179930]. Tucidinostat, an orally bioavailable iHDAC is now in phase III clinical trials [NCT04231448] in combined with R-CHOP regimen,. Administrating this combined treatment, compared to using the R-CHOP regimen alone, displayed a prolonged event‐free survival [741].

#### PI3K inhibitors

MYC half-life is less than 30 min and its translation significantly depends on the eukaryotic translation initiation factor 4 (eIF4) complex. The eIF4E-binding protein 1 (4E-BP1) sequestrates eIF4E, and following hyperphosphorylation of 4E-BP1, mRNA translation would be initiated as a result of multiple upstream signals. PI3K can independently phosphorylate of 4E-BP1, leading to translation of MYC mRNA [742]. Inhibition of PI3K by compounds such as Idelalisib [743], TGR-1202 [742], Fimepinostat (CUDC-907) [744], and BR101801 [745] can significantly reduce the MYC mRNA translation.

In contrast to Idelalisib, TGR-1202 not only inhibits PI3K but also targets casein kinase-1 ε (CK1ε). The mechanism of action of CK1ε in phosphorylating 4E-BP1 is similar to PI3K. Therefore, the dual inhibitory effect of TGR-1202 makes it clinically more potential than Idelalisib in the treatment of aggressive lymphoma [742]. Both compounds have now entered clinical trials.

The effectiveness of PI3K inhibitors is limited by the parallel activation of other survival-supporting pathways leading to drug resistance [746]. CUDC-907, a dual inhibitor of PI3Kδ and HDACs seems to be able to surpass the limitation of the inhibitors that only targets PI3K [747]. CUDC-907 has been tested in clinical trials on various hematological cancers, including relapsed or refractory lymphoma, multiple myeloma, MYC-altered DLBCL, and CLL. The results showed tolerability, safety, and efficiency [744, 748, 749].

DNA-activated protein kinase (DNA-PK) is a PI3K-related kinase capable of phosphorylating MYC at multiple serine residues, promoting its oncogenic activity in MYC-driven B-cell lymphomas [750, 751]. BR101801 is a first-in-class, orally bioavailable small-molecule capable of targeting both DNA-PK and PI3Kδ. The dual inhibitory mechanism of BR101801 in double-hit lymphoma cells, downregulates the MYC stability regardless of its translocation, amplification, and overexpression. This has led to significant growth inhibition in double-hit and double expressing DLBCL cells. Moreover, the combination of BR101801 with Venetoclax synergically enhances its antitumor effects, highlighting the great potential of this treatment approach [745, 752].

#### CDK inhibitors

CDK9, part of the positive transcription elongation factor b (p-TEFb) complex is essential for phosphorylation of a serine residue at CTD of RNA polymerase II recruited by MYC [753, 754]. MYC binding to p-TEFb activates RNA polymerase II and initiates transcription. This process has been shown to be critical for maintenance of MYC-driven model of hepatocellular carcinoma [145, 753, 755].

Dinaciclib inhibits kinase ability of CDK1, CDK2, CDK5 and CDK9 in a dose dependent manner. It is now in phase I/II clinical trials for different cancers, including both solid tumors and hematological cancers [756]. Investigation of Dinaciclib-mediated inhibition of CDK9 in aggressive MYC-induced lymphomas demonstrated the remarkable effectiveness of CDK9 inhibitors [757]. Of note, that Dinaciclib reduced the anti-apoptotic factor MCL-1 [757].

TG02 is a CDK inhibitor capable of inhibiting CDK1, CDK2, CDK7, CDK9, JAK2 and FLT3. This multi kinase inhibitor not only inhibits MYC by targeting CDKs but also inhibits BCR-signaling mediators, which could lead to further MYC inhibition and higher antitumor activity [758, 759]. Due to promising results from studies investigating the effects of TG02 on hematological malignancies [758, 759], this agent has entered clinical trials. [NCT01204164].

The most novel ultra-selective CDK9 inhibitor KB-0742, an orally bioavailable inhibitor, has displayed great antitumor potential in pre-clinical investigation [760]. The remarkable results from KB-0742 have led to clinical trials investigating relapsed and refractory solid tumors, and non-Hodgkin lymphoma [NCT04718675].

#### G-quadraplex stabilizers

The nuclease hypersensitivity element III1 (NHE III1) located at the MYC promoter controls 80–90% of the transcriptional activity of MYC gene. A particular site of NHE III1 creates G-quadruplex acting as a silencer region [761]. It makes this specific region a great target for drugs, stabilizing G-quadruplex.

In general, G-quadruplex stabilization can cause DNA double-strand breaks and promote apoptosis [762]. However, compounds such as CX-3543 can stabilize G-quadruplex of MYC promoter, selectively [763]. This small molecule also interrupts nucleolin/rDNA G-quadruplex formation, subsequently causing apoptosis. CX-3543 was the first G-quadruplex stabilizer to enter clinical trials [764].

APTO-253, a small molecule that regulates CDKN1A (p21), is capable of propelling cell-cycle arrest and triggering apoptosis in AML. Further investigations revealed that APTO-253 decreases the MYC mRNA translation and reduced the MYC levels. The underlying mechanism was found to be the APTO-253-induced G-quadruplex stabilization [765]. AML cells can convert the monomeric APTO-253 into Fe(253)_3_. Both APTO-253 and its ferrous form are capable of inducing G-quadruplex stabilization at promoters of MYC, and KIT [765]. Due to its promising potential, APTO-253 is now being clinically evaluated in AML and high-risk myelodysplasia patients [NCT02267863].

## Conclusion and future perspectives

MYC's twisted biology, particularly in hematopoiesis, has been comprehensively elucidated in recent years. We now realize that MYC facilitates the cancer cells' machinery. Blood malignancies are not an exception. Studies have shown that even temporary inactivation of MYC abrogates tumor progression, implying that MYC regulation could be a potential strategy to treat MYC-involved cancers [766, 767]. However, direct aiming for MYC is challenging. MYC does not possess a specific active site to be targeted by small molecules. This makes it hard to inhibit. Moreover, MYC is mainly found in the nucleus; therefore targeting MYC with antibodies is not feasible [1]. The broad MYC-mediated biological functions, vital to cells, also make it hard to completely eliminate. Future clinical studies will have to evaluate whether MYC should be targeted directly or indirectly in order to achieve a proper therapeutic outcome.

In the era of personalized medicine, the development of gene editing tools such as CRISPR/CAS9, and viral and non-viral based gene therapies have shown to be very promising [768]. As such MYC could be a promising choice for gene manipulation approaches.

## Data Availability

Not applicable.

## Abbreviations

BR: basic-region
HLH: helix-loop-helix
LZ: leucine-zipper
MBs: MYC boxes
DDR: DNA damage response
BM: bone marrow
ALL: acute lymphoblastic leukemia
CLL: chronic lymphocytic leukemia
NFAT: nuclear factor of activated T cells
NFAT1-NFAT4: family of transcription factors includes four Ca^2+^-regulated members
PTM: post-translational modifications
TAD: transactivation domain
ERK: extracellular receptor kinase
GSK-3β: glycogen synthase kinase
BRD4: bromodomain protein 4
HAT: histone acetyltransferase
MAPK: mitogen-activated protein kinase
PP2A: protein phosphatase 2A
wt: wild type
CKIs: CDK inhibitory proteins
ORC: origin recognition complex
PTTG1: securin gene expression
lncRNA: long-non coding RNA
MINCR: MYC-induced long noncoding RNA
YB1: Y box binding protein
BIN1: bridging integrator 1
AIF: apoptosis-inducing factor
TNFR: tumor necrosis factor receptor
MPPs: multipotent progenitors
CMPs and CLPs: common myeloid and lymphoid progenitors
BCR-ABL1: B-cell receptor–ABL proto-oncogene 1
DLBCL: diffuse large B-cell lymphoma
MM: multiple myeloma
MAX: MYC-associated protein X
TKIs: tyrosine kinase inhibitors
BENC: blood enhancer cluster
