# Correction to: MYC: a multipurpose oncogene with prognostic and therapeutic implications in blood malignancies

**DOI:** 10.1186/s13045-021-01152-9

**Published:** 2021-09-03

**Authors:** Seyed Esmaeil Ahmadi, Samira Rahimi, Bahman Zarandi, Rouzbeh Chegeni, Majid Safa

**Affiliations:** 1 Department of Hematology and Blood Banking, Faculty of Allied Medicine, Iran University of Medical Sciences, Tehran, Iran; 2 Medical Laboratory Sciences Program, College of Health and Human Sciences, Northern Illinois University, DeKalb, IL USA; 3 Cellular and Molecular Research Center, Iran University of Medical Sciences, Tehran, Iran

## Correction to: J Hematol Oncol (2021) 14:121 10.1186/s13045-021-01111-4

The original article [1] incorrectly states that OMO-1 is a MYC inhibitor in two instances: in the Direct MYC Inhibition sub-section of the MYC Inhibitors section, and in Table 3.

The authors wish to clarify that OMO-1 is not a MYC inhibitor, and its mentions in the original article should be disregarded.
